# Hepatoprotective Formula 919 ameliorates hepatic steatosis and metabolic disturbance in NAFLD with involvement of AMPK signaling

**DOI:** 10.3389/fphar.2026.1892654

**Published:** 2026-09-28

**Authors:** Manman Chen, Zheng Xu, Yu Chen, Yuemei Xu, Lan Zhang, Yiling Zhao, Pengfei Gao

**Affiliations:** 1 Department of Traditional Chinese Medicine, Jinshan Hospital, Fudan University, Shanghai, China; 2 Future Network Center, Purple Mountain Laboratories, Nanjing, China; 3 Department of Traditional Chinese Medicine, Shanghai Xuhui Central Hospital, Zhongshan-Xuhui Hospital, Fudan University, Shanghai, China

**Keywords:** AMPK signaling, hepatic steatosis, Hepatoprotective Formula 919, lipid metabolism, metabolic disturbance, NAFLD

## Abstract

**Background:**

Non-alcoholic fatty liver disease (NAFLD) is characterized by hepatic lipid accumulation and metabolic dysregulation, while effective therapeutic options remain limited. Hepatoprotective Formula 919 (HF919), a traditional Chinese herbal formulation, has shown hepatoprotective effects, but its mechanisms in NAFLD remain incompletely understood.

**Objective:**

To evaluate the effects of HF919 on hepatic steatosis and metabolic disturbance in NAFLD and the possible involvement of AMPK-associated metabolic regulation.

**Methods:**

Candidate absorbed constituents of HF919 were characterized by UPLC-HRMS and prioritized by *in silico* screening based on predicted oral bioavailability and drug-likeness. Network pharmacology, public human liver transcriptomic analyses, and molecular docking were integrated to examine disease-relevant candidate targets and pathways. The *in vivo* effects of HF919 were evaluated in a high-fat diet-induced NAFLD rat model using histological, biochemical, molecular, and immunohistochemical analyses. Fecal short-chain fatty acid (SCFA) profiling was additionally performed as an exploratory metabolic analysis.

**Results:**

HF919 alleviated hepatic steatosis, reduced hepatic lipid accumulation, and improved serum and hepatic lipid-related indices in HFD-induced NAFLD rats. HF919 treatment increased hepatic p-AMPK abundance and *Ppara* expression and reduced the expression of *Tlr4, Nfkb2,* and *Tgfb1*. Hepatic F4/80-positive staining was significantly reduced, accompanied by increased expression of the macrophage-associated genes *Mertk* and *Arg1*. Public liver transcriptomic analyses showed lower AMPK- and fatty-acid-oxidation-associated transcriptional features in NASH and in more severe histopathological categories, supporting the disease relevance of these metabolic alterations. Integration of HF919-predicted targets with disease-related transcriptomic changes identified metabolic and injury-related candidate pathways, while molecular docking showed favorable predicted docking scores for selected ligand–target pairs. Exploratory SCFA analysis showed shifts in several fecal SCFAs toward the control-associated pattern and associations with selected hepatic metabolic and molecular parameters.

**Conclusion:**

HF919 ameliorated hepatic steatosis and metabolic abnormalities in HFD-induced NAFLD rats. The accompanying increase in hepatic p-AMPK abundance and *Ppara* expression, together with disease-context evidence from human liver transcriptomic datasets, supports the possible involvement of AMPK-associated metabolic regulation. Changes in selected injury-related and macrophage-associated markers and fecal SCFA profiles provided additional secondary or exploratory observations. Further mechanistic studies are needed to establish the causal contribution of AMPK signaling to the effects of HF919.

## 1 Introduction

Over the past decades, the global rise in obesity, type 2 diabetes, and metabolic syndrome has been accompanied by a marked increase in non-alcoholic fatty liver disease (NAFLD) (Williams et al., 2011). As the hepatic manifestation of systemic metabolic imbalance, NAFLD is now recognized as one of the most prevalent chronic liver diseases worldwide (Younossi et al., 2023). NAFLD encompasses a spectrum ranging from simple steatosis to non-alcoholic steatohepatitis (NASH), with a subset of patients progressing to fibrosis, cirrhosis, and hepatocellular carcinoma (Friedman et al., 2018). Given its high prevalence and progressive nature (Powell et al., 2021), preventing the transition from hepatic steatosis to more advanced liver injury has become a major challenge in hepatology.

A central pathological feature of NAFLD is hepatic lipid metabolic imbalance, characterized by excessive lipid deposition, impaired fatty acid oxidation, and enhanced lipogenesis (Zhao and Saltiel, 2020). AMP-activated protein kinase (AMPK) is a key cellular energy sensor involved in the regulation of hepatic lipid homeostasis by promoting fatty acid oxidation and restraining lipogenic drive under metabolic stress (Cusi et al., 2021). Reduced AMPK activity has been reported in NAFLD and is associated with hepatic steatosis and metabolic dysfunction (Smith et al., 2016; Garcia et al., 2019). Accordingly, AMPK signaling has attracted considerable interest as a potential therapeutic target in the treatment of NAFLD.

In addition to intrahepatic metabolic dysregulation, gut-derived metabolites such as short-chain fatty acids (SCFAs) have been implicated in systemic metabolic regulation and NAFLD pathophysiology (Ding et al., 2019; Zhang et al., 2021). SCFAs may influence hepatic energy balance and lipid metabolism (Shashni et al., 2023; Li et al., 2025), raising the possibility that changes in hepatic metabolic status may occur alongside alterations in fecal SCFA profiles. However, the relationship between these metabolic changes in NAFLD remains incompletely understood.

Traditional Chinese medicine (TCM) has long been used in the management of chronic liver diseases and may offer advantages through multi-component and multi-target regulation (Wang et al., 2025; Zhang et al., 2024). Hepatoprotective Formula 919 (HF919), a traditional herbal preparation developed on the basis of Chinese medicinal theory and protected by a Chinese invention patent (CN107929413B), has shown hepatoprotective effects in previous studies (Huang et al., 1995). Preliminary HPLC/ESI-MS analysis has revealed that HF919 contains multiple bioactive constituents, including diterpenoids, lignans, flavonoids, and phenolic acids (Xu et al., 2017). Pharmacological studies have suggested that HF919 protects hepatocytes, promotes hepatic tissue repair (Gao and Zhang, 2005), exhibits anti-hepatitis B virus activity (Huang and Gao, 2002), and attenuates hepatic steatosis (Chen et al., 2020) and fibrosis (Huang et al., 2003). Previous work has also indicated anti-inflammatory effects of HF919, including suppression of NF-κB-related signaling (Chen et al., 2020), as well as potential regulatory effects on gut microbial disturbance (Chen et al., 2021). However, the molecular basis underlying the anti-steatotic effects of HF919 remains incompletely understood, particularly whether AMPK-associated metabolic regulation is involved.

In the present study, we investigated the effects of HF919 on hepatic steatosis and metabolic disturbance in NAFLD, with particular attention to the possible involvement of AMPK-associated metabolic regulation. UPLC-HRMS, network pharmacology, public human liver transcriptomic analyses, and molecular docking were integrated to characterize candidate HF919 constituents and examine disease-relevant targets and pathways. The *in vivo* effects of HF919 were subsequently evaluated in an HFD-induced NAFLD rat model using histological, biochemical, and molecular analyses, with fecal SCFA profiling included as an exploratory metabolic assessment. By integrating computational, transcriptomic, and experimental evidence, this study aimed to clarify the metabolic effects of HF919 in NAFLD and assess the possible involvement of AMPK-associated regulation.

## 2 Materials and methods

### 2.1 Experimental design

The study was designed to evaluate the effects of HF919 on hepatic steatosis and metabolic disturbance in HFD-induced NAFLD rats and to explore the possible involvement of AMPK-associated regulation. Candidate absorbed constituents of HF919 were characterized by UPLC-HRMS and subsequently prioritized using *in silico* screening based on predicted oral bioavailability and drug-likeness. Network pharmacology and public liver transcriptomic analyses were used to identify disease-relevant candidate targets and pathways, and representative ligand-target pairs were subsequently evaluated by molecular docking. The *in vivo* effects of HF919 were assessed by histological, biochemical, molecular, immunohistochemical, and targeted fecal SCFA analyses.

### 2.2 Preparation of HF919

HF919 is a traditional Chinese herbal formulation composed of multiple medicinal herbs, including *Actinidia chinensis* Planch. (20 g), *Salvia miltiorrhiza* Bunge (10 g), *Atractylodes macrocephala* Koidz. (5 g), *Schisandra chinensis* (Turcz.) Baill. (5 g), and *Citrus reticulata* Blanco (2.5 g), among other components. The formulation was provided by Shanghai Wanshicheng Guoyao Products Co., Ltd. (Shanghai, China) according to a standardized preparation process. The final concentration of HF919 was 1.35 g/mL. The dosage used in this study (10 mL/kg/day) was determined based on previous studies (Huang et al., 1995; Huang and Gao, 2002; Xu et al., 2017; Chen et al., 2020; Chen et al., 2021) and converted from clinical dosage using body surface area normalization.

### 2.3 Preparation of HF919 medicated serum

To identify candidate absorbed constituents of HF919, medicated serum was prepared in Sprague-Dawley rats. Ten rats were initially allocated to the medicated-serum experiment and assigned to the HF919 and control groups. Four animals died during the gavage period before serum collection. Serum samples from the six surviving animals (n = 3 biological replicates per group) were therefore included in the UPLC-HRMS analysis.

The HF919 group received HF919 at 10 mL/kg by intragastric gavage twice daily for 3 consecutive days, whereas the control group received an equal volume of saline. One hour after the final administration, rats were deeply anesthetized with sodium pentobarbital (50 mg/kg, i. p.), and blood was collected from the abdominal aorta. After clotting at room temperature for 30 min, blood samples were centrifuged at 3,000 rpm for 15 min at 4 °C. The resulting serum was heat-inactivated at 56 °C for 30 min, filtered through a 0.22-μm membrane, and stored at −80 °C until analysis. Serum samples were processed and analyzed individually and were not pooled.

### 2.4 UPLC-HRMS analysis

The chemical constituents of HF919, medicated serum, and control serum were analyzed using ultra-performance liquid chromatography coupled with high-resolution mass spectrometry (UPLC-HRMS). Chromatographic separation was performed on a Waters ACQUITY UPLC BEH C18 column (2.1 × 100 mm, 1.7 μm) using acetonitrile and water containing 0.1% formic acid as the mobile phases. The gradient program was as follows: 5% acetonitrile at 0 min, linearly increased to 98% at 17.0 min, returned to 5% at 17.2 min, and maintained until 20.0 min. The flow rate was 0.3 mL/min, and the column temperature was maintained at 35 °C.

Mass spectrometric analysis was performed using a Q-Exactive HFX high-resolution mass spectrometer (Thermo Fisher Scientific, Germany) equipped with an electrospray ionization (ESI) source operating in both positive- and negative-ion modes. Data were acquired using a data-dependent acquisition (DDA) strategy over an MS1 scan range of *m/z* 90–1,300. The 10 most intense precursor ions in each MS1 scan were automatically selected for MS/MS analysis. Fragmentation was performed using stepped higher-energy collisional dissociation (HCD) with normalized collision energies of 20, 40, and 60. The capillary temperature and probe heater temperature were set at 320 °C and 370 °C, respectively.

Compound annotation was based on accurate precursor mass, isotopic distribution, retention time, adduct type, and MS/MS fragment information, with database-assisted matching against HMDB (Wishart et al., 2022), METLIN (Smith et al., 2005), and MassBank (Horai et al., 2010). Candidate annotations were required to have an MS1 mass error of <15 ppm, with MS/MS spectral information used to support structural assignment where available. Peak-area signals were used to assess the relative abundance of detected compounds. Components detected in HF919 and medicated serum but absent from control serum were considered candidate absorbed constituents. For the five representative absorbed constituents reported in this study, signals were consistently detected in all three medicated-serum samples but were absent from all three control-serum samples. The absorbed constituents were subsequently screened using the Traditional Chinese Medicine Systems Pharmacology database (TCMSP, v2.3), and compounds meeting the predefined criteria of oral bioavailability (OB) ≥30% and drug-likeness (DL) ≥0.18 were retained for subsequent network pharmacology analyses.

### 2.5 Network pharmacology analysis

#### 2.5.1 Collection of drug and disease targets

Potential human targets of the selected candidate absorbed constituents of HF919 were retrieved from the Traditional Chinese Medicine Systems Pharmacology database (TCMSP, https://tcmsp-e.com/tcmsp.php), Comparative Toxicogenomics Database (CTD, https://ctdbase.org//), PharmMapper (https://www.lilab-ecust.cn/pharmmapper/), and SwissTargetPrediction (https://www.swisstargetprediction.ch/). NAFLD-related targets were collected from GeneCards (https://www.genecards.org/), OMIM (https://omim.org/), PharmGKB (https://www.pharmgkb.org/), Therapeutic Target Database (TTD, https://db.idrblab.net/ttd/), DrugBank (https://go.drugbank.com/), and DisGeNET (https://www.disgenet.org/). Only human targets were retained. Target identifiers were converted to official gene symbols, and duplicated entries were removed before merging the results from the individual databases. The database-specific search entities, target-selection and processing criteria, and access information are provided in Supplementary Table S1.

#### 2.5.2 Identification of overlapping and core targets

HF919-related and NAFLD-related target sets were intersected using Venny 2.1 to identify candidate overlapping targets. The overlapping targets were imported into STRING (v12.0), with the organism restricted to *Homo sapiens* and a minimum required interaction score of 0.400, to construct the protein–protein interaction (PPI) network. Network topology was analyzed using Cytoscape (v3.10.3) with the CentiScaPe plugin. Degree, Closeness, and Betweenness centrality were calculated. The mean values of each centrality metric across all nodes were used as cutoff thresholds. Nodes with degree, closeness, and betweenness values greater than or equal to the corresponding thresholds (8.1429, 0.0151, and 34.5143, respectively) were defined as core targets.

#### 2.5.3 Functional enrichment analysis

Gene Ontology (GO) and Kyoto Encyclopedia of Genes and Genomes (KEGG) enrichment analyses of the candidate overlapping targets were performed in R (version 4.3.2) using clusterProfiler (version 4.10.0). GO enrichment was evaluated separately for biological process, cellular component, and molecular function categories. Terms and pathways meeting the prespecified criteria of *P* < 0.05 and *q* < 0.05 were retained as significant. The top-ranked GO terms and the 30 highest-ranked KEGG pathways were displayed for visualization. A compound–target–pathway network was subsequently constructed to visualize the relationships among the selected candidate HF919 constituents, candidate targets, and enriched pathways.

### 2.6 Public transcriptomic analysis

Public human liver transcriptomic datasets GSE126848 and GSE130970 were obtained from the NCBI Gene Expression Omnibus (GEO) database. GSE126848 was used for cross-sectional analysis of disease-associated transcriptional changes, whereas GSE130970 was used to examine transcriptional features across histopathological severity categories. Dataset platforms, sample composition, expression-matrix sources, and preprocessing procedures are summarized in Supplementary Table S2.

For GSE126848, differential-expression analysis was performed using the limma package (v3.54.2) in R. The Control vs. NASH comparison served as the primary analysis in the main text, whereas the Control vs. NAFLD comparison was performed in parallel and is presented in Supplementary Figure S1. P values from differential-expression analysis were adjusted using the Benjamini–Hochberg method, and DEGs were defined according to the prespecified thresholds of |log2FC| > 1 and adjusted *P* < 0.05. Sample-level AMPK-related and FAO-protective scores were calculated using single-sample gene set enrichment analysis (ssGSEA). Differences in pathway scores and selected gene expression between two groups were evaluated using the Wilcoxon rank-sum test. KEGG gene set enrichment analysis was performed using clusterProfiler based on log2 fold-change–ranked gene lists, with an adjusted *p*-value (FDR) < 0.05 used to define significantly enriched pathways.

For GSE130970, the AMPK activation-associated signature and selected injury-related genes were compared across histopathological severity categories, including NAFLD activity score, steatosis grade, hepatocellular ballooning grade, lobular inflammation grade, and fibrosis stage. Comparisons across severity groups were performed using the Kruskal–Wallis test. These analyses were interpreted as group-wise differences across pathological categories rather than as evidence of a causal relationship.

To evaluate the disease relevance of HF919-predicted targets, the HF919 target set was integrated with DEGs derived from both the Control vs. NASH and Control vs. NAFLD comparisons in GSE126848. Genes shared among the HF919-predicted targets, NASH-related DEGs, and NAFLD-related DEGs were retained for subsequent GO, KEGG, and PPI analyses. This integrative analysis was used to identify disease-relevant overlap between pharmacologically predicted HF919 targets and transcriptional alterations in human liver tissue and was not interpreted as evidence that HF919 directly regulates these genes in human NAFLD.

### 2.7 Molecular docking

Molecular docking was performed to evaluate the potential binding of the major candidate compounds of HF919 to representative target proteins identified from the network pharmacology and transcriptomic-integrative analyses. AMPK, TGFB1, and ARG1 were selected as representative targets because of their relevance to AMPK-associated metabolic regulation, injury-related signaling, and macrophage-associated changes, respectively.

Three-dimensional structures of Cryptotanshinone, Licocoumarone, Schizandrin, Skullcapflavone II, and Hydroxytanshinone were retrieved from PubChem and prepared using AutoDockTools (v1.5.7) by adding hydrogen atoms and assigning Gasteiger charges. Protein structures were obtained from the Protein Data Bank and prepared by removing non-target protein chains, crystallographic water molecules, and co-crystallized ligands using PyMOL (v2.5.0), followed by hydrogen addition and charge assignment in AutoDockTools. Ligands and receptors were converted to PDBQT format. PDB IDs, retained receptor structures, and receptor-specific docking-grid parameters are provided in Supplementary Table S3.

Docking simulations were performed using AutoDock Vina (v1.2.3) with receptor-specific search spaces, an exhaustiveness of 40, an energy range of 5 kcal/mol, and a maximum of 10 binding modes for each ligand–receptor pair. Docking poses were ranked according to the Vina docking score, and the pose with the most negative score for each ligand–receptor pair was selected for visualization and inspection of predicted ligand–residue contacts using PyMOL (v2.5.0). Docking results were interpreted as computational predictions of potential ligand–target compatibility rather than evidence of physical binding or functional target engagement.

### 2.8 Animals and NAFLD model

A total of 40 male Sprague–Dawley rats (5 weeks old, 150 ± 15 g) were obtained from Shanghai Bikai Keyi Biotechnology Co., Ltd. (Shanghai, China; animal production license no. SCXK 2018-0006) and housed under specific pathogen-free (SPF) conditions (22 °C ± 2 °C, 50%–60% humidity, 12 h light/dark cycle). All experimental procedures were approved by the Animal Welfare and Ethics Committee of the Department of Laboratory Animal Science, Fudan University, and were conducted in accordance with institutional guidelines.

Of the 40 rats, 10 were initially allocated to the medicated-serum experiment. Four animals died during the gavage period before the final serum collection, leaving six rats for the final medicated-serum analysis (n = 3 per group), as described in Section 2.3. The remaining 30 rats were allocated to the Control, NAFLD, and HF919 groups (n = 10 per group). Rats in the Control group were maintained on a standard diet, whereas rats in the NAFLD and HF919 groups received a high-fat diet (HFD) for 16 weeks. The HFD consisted of standard chow supplemented with 10% lard, 3% sesame oil, 2% cholesterol, 0.5% sodium cholate, 20% fructose, 12% casein, 2% vitamins, and 2% minerals, providing approximately 40% of total energy from fat and 53.3% from carbohydrates. Histopathological examination at the end of the experiment confirmed hepatic steatosis in the untreated NAFLD group.

### 2.9 Treatment protocol

Beginning at week 17, the HFD was discontinued, and all rats were maintained on a standard diet during the 4-week intervention period. Rats in the HF919 group received HF919 (10 mL/kg/day) by intragastric gavage for 4 weeks, whereas rats in the Control and NAFLD groups received an equivalent volume of saline. Body weight and food intake were monitored throughout the experiment. At the end of the intervention, rats were deeply anesthetized with sodium pentobarbital (50 mg/kg, i. p.). Blood was collected by cardiac puncture under deep anesthesia, and euthanasia was completed by exsanguination. Liver and fecal samples were subsequently collected for further analyses.

Biological replicate numbers differed among downstream analyses according to sample allocation and sample availability. The liver-to-body weight ratio was evaluated in six rats per group; serum biochemical measurements in eight rats per group; hepatic TG and TC measurements in six rats per group; qRT-PCR and Western blotting in three rats per group; H&E staining, Oil Red O staining, and F4/80 immunohistochemistry in three rats per group; and fecal SCFA profiling in six rats per group. The exact biological replicate number for each analysis is also indicated in the corresponding figure legend.

### 2.10 Histological analysis

For H&E staining, liver tissues were fixed in 4% paraformaldehyde for 24 h, dehydrated, cleared, embedded in paraffin, and sectioned at a thickness of 5–7 μm. After deparaffinization and rehydration, the sections were stained with hematoxylin and eosin using a standard procedure. Liver histopathology was examined and photographed using an Olympus BX51 light microscope (Olympus, Japan) at ×200 magnification. Three independent rats per group were examined.

For Oil Red O staining, frozen liver tissues were sectioned at a thickness of 5–7 μm. After air-drying at room temperature, the sections were fixed in 4% paraformaldehyde for 10 min, rinsed with distilled water and 60% isopropanol, and stained with freshly prepared Oil Red O working solution for 10 min. Excess stain was removed with 60% isopropanol, followed by rinsing with distilled water. The sections were counterstained with Mayer’s hematoxylin for 40 s, blued under running tap water, and mounted with glycerol gelatin. Images were acquired at ×100 magnification. Oil Red O-positive lipid droplets were quantified using ImageJ 1.54v software from three independent rats per group. Particle count, total Oil Red O-positive area, average particle size, and percentage positive area (%Area) were recorded. Scale-bar and annotation regions were excluded from the image-analysis region of interest. Image-analysis settings were applied consistently across experimental groups.

### 2.11 Immunohistochemistry (IHC)

Liver tissues were fixed in 4% paraformaldehyde, embedded in paraffin, and sectioned at approximately 5 μm. After deparaffinization and rehydration, endogenous peroxidase activity was blocked with 3% hydrogen peroxide for 10 min. Antigen retrieval was performed in citrate buffer (pH 6.0) using heat-mediated retrieval. Sections were washed with PBS and blocked with 5% bovine serum albumin for 30 min, followed by overnight incubation at 4 °C with an anti-F4/80 primary antibody (Novus Biologicals, NBP3-33457). After washing, the sections were incubated with a goat anti-rabbit IgG (H + L) HRP-conjugated secondary antibody (Novus Biologicals, NBP1-75325**)** for 1 h at room temperature. Immunoreactivity was visualized using DAB, and the sections were counterstained with hematoxylin, dehydrated, cleared, and mounted with neutral balsam.

Images were acquired at ×200 magnification. F4/80-positive staining was quantified using ImageJ in three independent rats per group. The percentage of F4/80-positive staining area (%Area) was used for group-level quantification; particle count, total positive area, and average positive-object size were additionally recorded.

### 2.12 Biochemical assays

Serum levels of triglycerides (TG), total cholesterol (TC), low-density lipoprotein cholesterol (LDL-C), high-density lipoprotein cholesterol (HDL-C), free fatty acids (FFA), alanine aminotransferase (ALT), and aspartate aminotransferase (AST) were measured using an automatic biochemical analyzer (Mindray BS-420) with commercial enzymatic kits according to the manufacturers’ instructions. For hepatic lipid measurements, approximately 0.1 g of liver tissue was homogenized on ice in 1 mL of the corresponding extraction solution. After centrifugation, hepatic TG and TC contents were determined using commercial assay kits (Solarbio, Beijing, China; TG, BC5945; TC, BC1985) according to the manufacturer’s instructions. Results were normalized to wet tissue weight and expressed as mmol/g tissue.

### 2.13 Quantitative real-time PCR (qRT-PCR)

Total RNA was extracted from liver tissue from three independent rats per group using the RNeasy Mini Kit (QIAGEN, Germany) according to the manufacturer’s instructions. Complementary DNA was synthesized using the ImProm-II™ Reverse Transcription System (Promega, United States). Quantitative real-time PCR was performed using an Mx3005P Real-Time PCR System (Agilent Technologies, United States) with Brilliant III Ultra-Fast SYBR Green QPCR Master Mix (Agilent Technologies, Cat. No. 600882).

The genes analyzed included the lipid metabolism-related gene *Ppara*, the injury/inflammation-associated genes *Tlr4* and *Nfkb2*, the fibrosis-associated gene *Tgfb1*, and the macrophage-associated genes *Arg1* and *Mertk*. *Actb* was used as the reference gene. Primer sequences are provided in Table 1. The amplification conditions consisted of an initial denaturation at 95 °C for 3 min, followed by 40 cycles of denaturation at 95 °C for 5 s and annealing/extension at 60 °C for 20 s. A melting-curve analysis was subsequently performed to verify amplification specificity. Relative mRNA expression was calculated using the 2^−ΔΔCt^ method after normalization to *Actb*. Each biological sample was analyzed in technical triplicate.

**TABLE 1:** Sequences of primers used for quantitative real-time PCR.

Gene	Forward primer (5′–3′)	Reverse primer (5′–3′)
*Tgfb1*	CAT​GGA​GCT​GGT​GAA​ACG​GAA​G	GAC​TGG​CGA​GCC​TTA​GTT​TGG​AC
*Arg1*	TGG​AGA​CCA​CAG​TAT​GGC​AAT​T	GTG​AGC​ATC​CAC​CCA​AAT​GAC
*Mertk*	TCC​TGA​CTC​CAT​CAT​TGC​CTC​T	ATC​TTC​CCA​TTC​CTC​GTT​GC
*Ppara*	CAT​CTG​TCC​TCT​CTC​CCC​ACT​TG	CCG​AGG​GAC​TGA​GAA​ATC​TCT​TG
*Tlr4*	CCATTGCTGCCAACATC	GCCAGAGCGGCTACTCA
*Nfkb2*	AGC​ACA​GAT​ACC​ACT​AAG​ACG​CAC​C	TGA​CCA​GGG​AGA​TTC​GAA​CTG​TTC
*Actb*	CGT​AAA​GAC​CTC​TAT​GCC​AAC​A	GGA​GGA​GCA​ATG​ATC​TTG​ATC​T

### 2.14 Western blot (WB)

Total protein was extracted from liver tissues from three independent rats per group using RIPA lysis buffer supplemented with protease and phosphatase inhibitors, and protein concentrations were determined using a BCA protein assay kit (Beyotime, China). Equal amounts of protein (30 μg per lane) were separated by 12% SDS-PAGE and transferred onto PVDF membranes. After blocking with 5% non-fat milk, the membranes were incubated overnight at 4 °C with primary antibodies against phosphorylated AMPKα1/α2 (HUABIO, China; Cat. No. HA723603) and β-actin (Proteintech, China; Cat. No. 66009-1-Ig). The membranes were subsequently incubated with HRP-conjugated goat anti-rabbit IgG secondary antibody (Proteintech, China; Cat. No. SA00001-2).

Protein bands were visualized using an enhanced chemiluminescence (ECL) detection system (Thermo Fisher Scientific, United States) and quantified by densitometric analysis using ImageJ software. The densitometric intensity of phosphorylated AMPKα1/α2 was normalized to that of β-actin.

### 2.15 SCFA analysis

Fecal short-chain fatty acids (SCFAs) were quantified by gas chromatography–mass spectrometry (GC–MS). Approximately 30 mg of frozen fecal material was homogenized with 350 μL of an aqueous solution containing 6,6,6-d3-hexanoic acid as the internal standard. After extraction and centrifugation, 100 μL of the supernatant was acidified with 10 μL of 5 M HCl and extracted three times with anhydrous diethyl ether. The combined organic phase was dehydrated with anhydrous sodium sulfate, and 160 μL of the extract was derivatized with 8 μL of N,O-bis(trimethylsilyl)trifluoroacetamide (BSTFA) at room temperature for >8 h. GC–MS analysis was performed using an Agilent 8890 gas chromatograph coupled to an Agilent 7000D triple-quadrupole mass spectrometer (Agilent Technologies, United States) equipped with an HP-5ms capillary column (30 m × 0.25 mm, 0.25 μm). Helium was used as the carrier gas at 1.0 mL/min. The injection volume was 1 μL with a split ratio of 10:1, and the inlet temperature was 260 °C. The oven temperature was initially held at 40 °C for 2 min, increased to 150 °C at 15 °C/min and held for 1 min, and then increased to 300 °C at 30 °C/min and held for 5 min. Electron-impact ionization was performed at 70 eV, and data were acquired in selected-ion-monitoring (SIM) mode.

SCFAs were identified by comparison of retention times and characteristic ions with authentic standards and quantified using internal-standard-normalized peak responses and calibration curves. SCFA profiling was treated as an exploratory analysis of fecal metabolic changes associated with HF919 treatment. Six biological samples per group were analyzed (n = 6 per group). Spearman correlation analyses were restricted to matched individual animals for which both variables were available.

### 2.16 Statistical analysis

Unless otherwise specified, animal data are presented as mean ± standard deviation (SD). For the animal experiments, Control versus NAFLD and NAFLD versus HF919 were compared using two-tailed Welch’s *t*-tests in Microsoft Excel. Control versus HF919 was not included as a planned pairwise comparison. No additional multiplicity adjustment was applied to these two comparisons; therefore, the reported *P* values are unadjusted. Exploratory Spearman correlation analyses involving hepatic p-AMPK were performed in R, whereas correlations between fecal SCFAs and NAFLD-related parameters were analyzed using SPSS 26.0. For transcriptomic analyses, differential expression in GSE126848 was analyzed using limma with Benjamini–Hochberg adjustment. Two-group comparisons of ssGSEA scores and selected gene expression were performed using the Wilcoxon rank-sum test, whereas overall comparisons across histopathological severity groups in GSE130970 were performed using the Kruskal–Wallis test. KEGG gene set enrichment analysis was performed using clusterProfiler, with multiple testing controlled using the Benjamini–Hochberg method where applicable. *P* < 0.05 was considered statistically significant unless an adjusted *P* value or *q* value was specified for the corresponding analysis.

## 3 Results

### 3.1 Characterization of candidate absorbed constituents of HF919

UPLC-HRMS was used to characterize the chemical profiles of HF919, medicated serum, and control serum. Several compounds detected in HF919 were also detected in medicated serum but not in control serum and were therefore considered candidate absorbed constituents. Among these constituents, five compounds met the predefined *in silico* screening criteria of predicted oral bioavailability (OB ≥ 30%) and drug-likeness (DL ≥ 0.18): Cryptotanshinone, Licocoumarone, Schizandrin, Skullcapflavone II, and Hydroxytanshinone (Table 2). These compounds were retained as selected candidate constituents for subsequent network pharmacology analyses.

**TABLE 2:** UPLC-HRMS characteristics and predicted OB/DL of five candidate absorbed HF919 constituents.

No.	Compounds	PubChem CID	Formula	Ionization mode	MS1 (m/z)	MS2 (m/z)	OB	DL
1	Cryptotanshinone	160254	C19H20O3	[M + H]^+^	297.148	297.145, 279.139, 251.143, 254.094, 298.15	52.34	0.40
2	Licocoumarone	503731	C20H20O5	[M + H]^+^	341.138	341.139, 323.124, 279.1, 309.112, 311.125	33.21	0.36
3	Schizandrin	3001664	C24H32O7	[M + H]^+^	433.221	417.217, 386.197, 331.152, 371.176, 348.145	46.27	0.84
4	Skullcapflavone II	124211	C19H18O8	[M + H]^+^	375.107	375.105, 345.063, 360.083, 376.11, 167.069	69.51	0.44
5	Hydroxytanshinone	5318350	C19H18O4	[M + H]^+^	311.127	283.133, 311.125, 265.119, 237.126, 284.137	44.93	0.44

### 3.2 Network pharmacology predicts that HF919 targets metabolic and injury-related pathways relevant to NAFLD

#### 3.2.1 Screening of candidate targets and construction of the PPI network

The overall network pharmacology workflow is shown in Figure 1A. A total of 544 putative HF919-related targets were retrieved from TCMSP, CTD, PharmMapper, and SwissTargetPrediction, whereas 937 NAFLD-related targets were collected from GeneCards, PharmGKB, OMIM, TTD, DrugBank, and DisGeNET. Intersection of the two target sets yielded 35 candidate overlapping targets (Figure 1B). These targets were used to construct a STRING-based PPI network comprising 35 nodes and 142 edges (Figure 1C). Topological analysis using the CentiScaPe plugin in Cytoscape identified 10 representative core targets according to the Degree, Closeness, and Betweenness thresholds described in the Methods: RELA, TNF, PPARG, NR1I2, MTOR, PARP1, GPT, INS, TGFB1, and ARG1 (Figure 1D).

**FIGURE 1:**
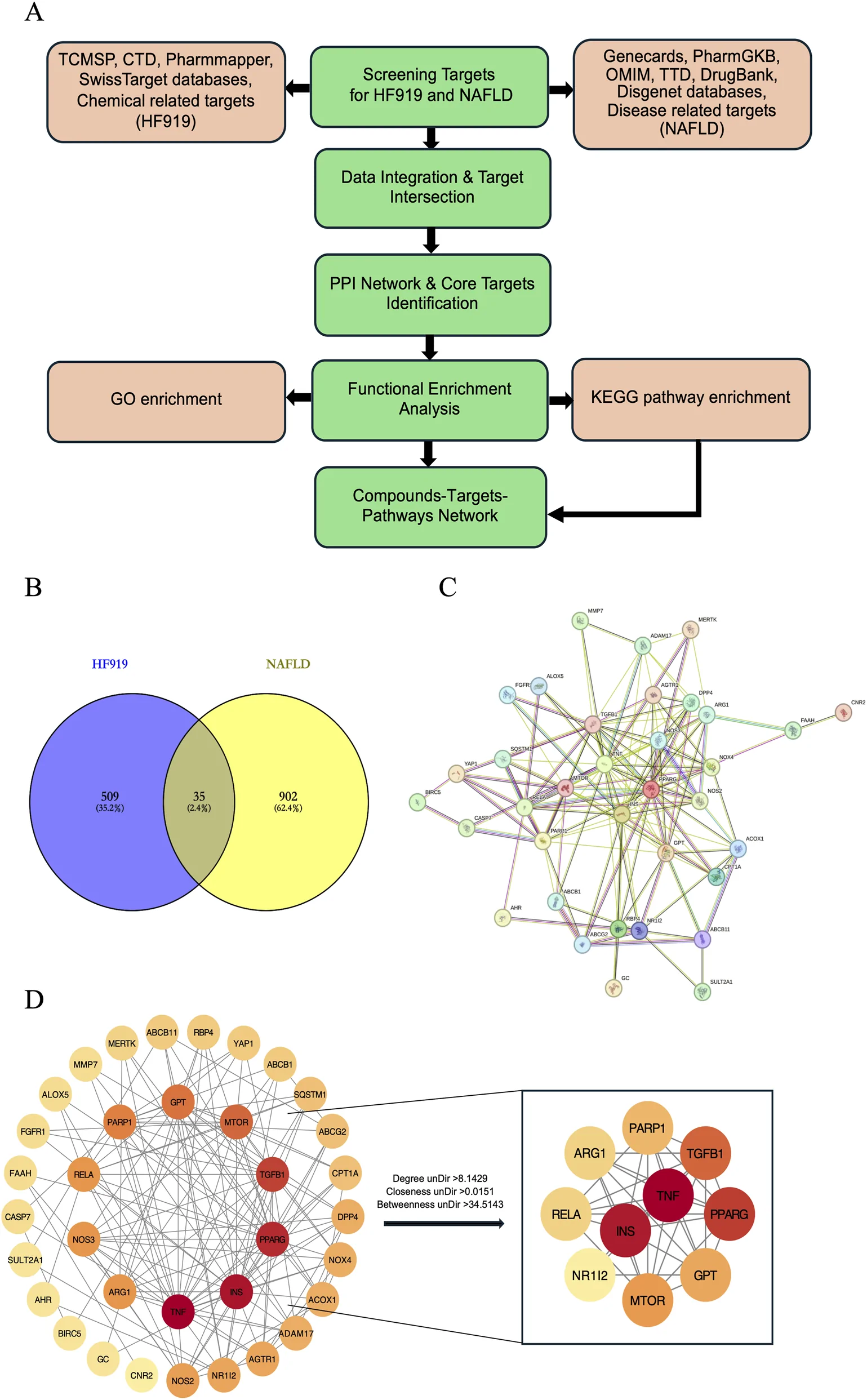
Network pharmacology workflow and candidate target identification of HF919 in NAFLD. **(A)** The workflow of network pharmacology analysis. **(B)** Venn diagram showing the overlap between HF919-related targets and NAFLD-related targets. **(C)** PPI network of the candidate targets. **(D)** Core targets identified by CentiScaPe-based topological analysis.

#### 3.2.2 Functional enrichment analysis of candidate targets

GO enrichment analysis indicated that the candidate targets were mainly enriched in the regulation of small-molecule metabolic processes, organic acid catabolism, wound healing, and responses to xenobiotic stimuli (Figure 2A). The enriched cellular component and molecular function terms further suggested involvement in membrane-associated signaling and ligand-activated transcriptional regulation. These enrichment results indicated that the candidate targets were functionally associated with metabolic processes and signal transduction.

**FIGURE 2:**
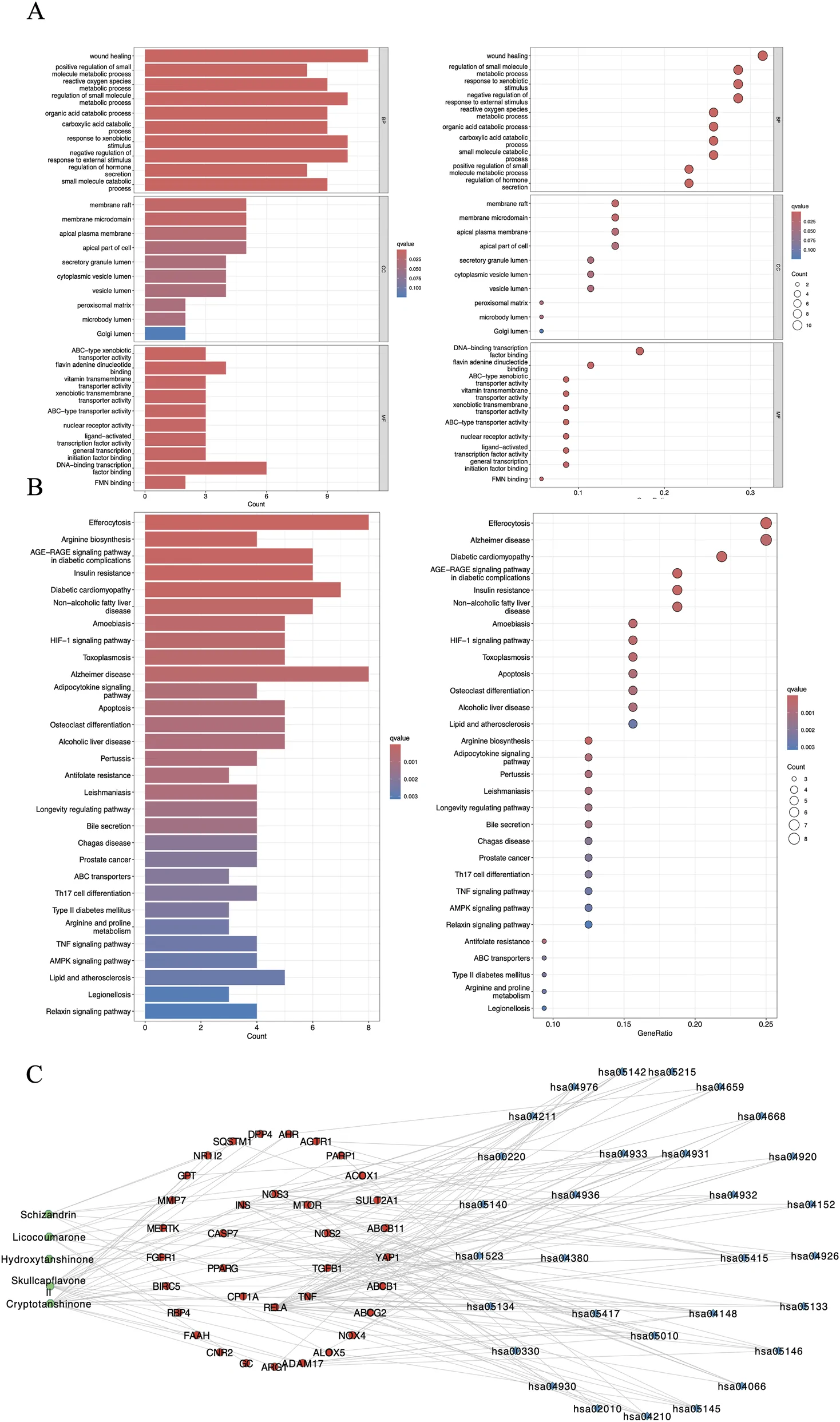
Functional enrichment analysis and the compounds-targets-pathways network. **(A)** GO enrichment analysis (BP, CC and MF terms) of the candidate targets. **(B)** KEGG pathway enrichment analysis of the candidate targets. **(C)** Compound–target–pathway network showing the relationships among the major HF919 compounds, candidate targets, and enriched pathways.

KEGG analysis showed enrichment of pathways relevant to NAFLD pathophysiology, including efferocytosis, NAFLD, lipid and atherosclerosis, AMPK signaling, TNF signaling, insulin resistance, and apoptosis (Figure 2B). A compound–target–pathway network was further constructed to visualize the predicted relationships among the five selected candidate HF919 constituents, candidate targets, and enriched pathways (Figure 2C).

Overall, the network pharmacology analysis identified candidate HF919-related targets and pathways associated with metabolic, inflammatory, and injury-related processes. Because these results were based on computational prediction, their disease relevance was subsequently examined using public liver transcriptomic datasets.

### 3.3 Public transcriptomic analyses identify AMPK-associated metabolic dysregulation across the NAFLD spectrum

#### 3.3.1 Cross-sectional transcriptomic analysis in GSE126848

To examine AMPK-associated metabolic alterations in human diseased liver, GSE126848 was analyzed using the Control vs. NASH comparison as the primary analysis. Differential expression analysis revealed substantial disease-associated transcriptional changes between control and NASH liver samples (Figures 3A,B). Both the AMPK-related score and the FAO-protective score were significantly lower in NASH samples than in controls (Figures 3C,D), accompanied by lower expression of *PPARA* (Figure 3E). Focused KEGG GSEA further identified negative enrichment of metabolism-related pathways, including AMPK-associated and fatty-acid-oxidation-related programs (Figure 3F). Consistent patterns were also observed in the Control vs. NAFLD comparison, as shown in Supplementary Figure S1. These findings indicate that human NAFLD/NASH liver exhibits reduced AMPK-associated and fatty-acid-oxidation-related transcriptional features.

**FIGURE 3:**
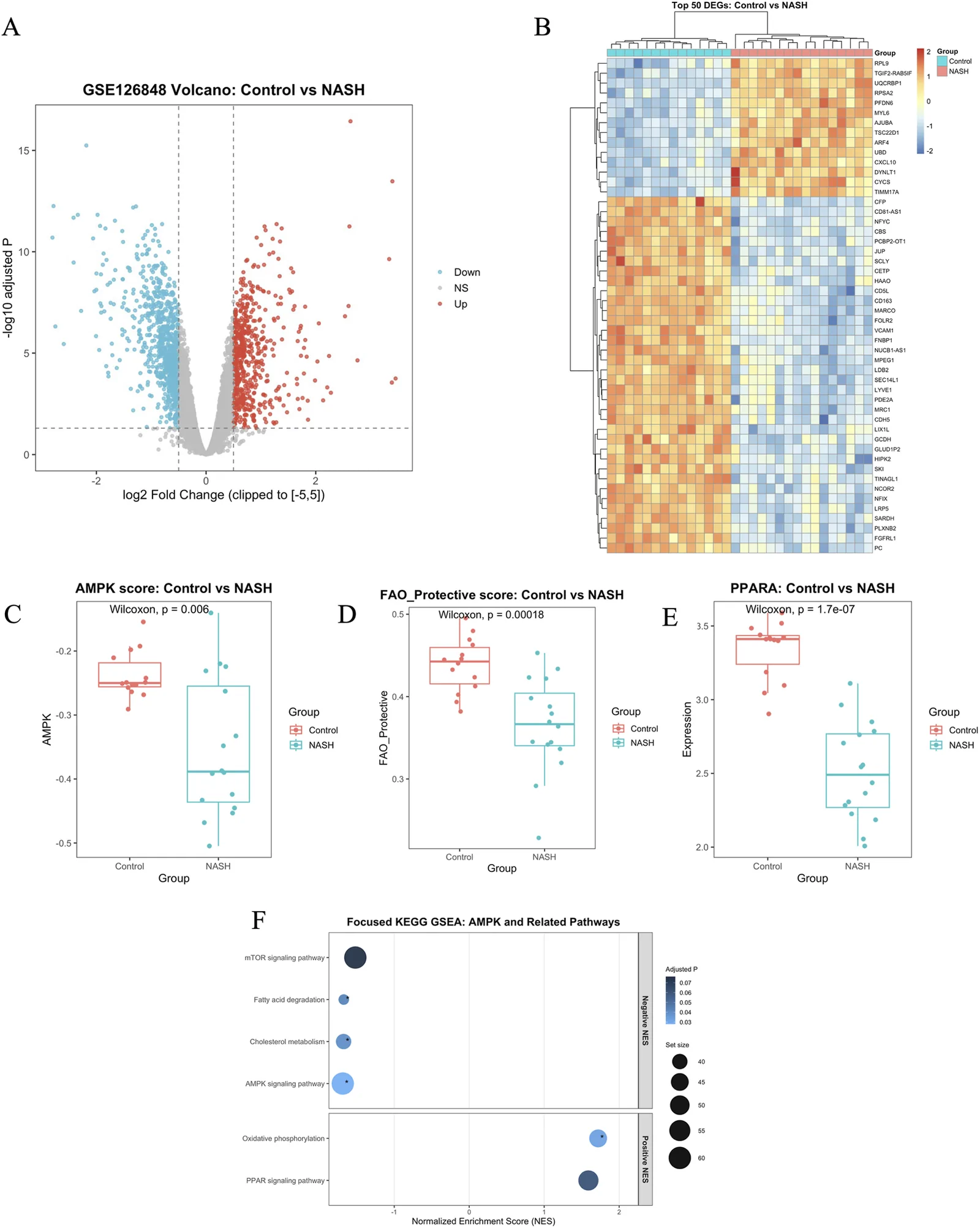
Cross-sectional transcriptomic validation in GSE126848 indicates suppression of AMPK-associated protective metabolic programs in diseased liver. **(A)** Volcano plot of differentially expressed genes between Control and NASH liver samples. **(B)** Heatmap of the top 50 differentially expressed genes. **(C,D)** Boxplots of the AMPK score and FAO-protective score. **(E)** Boxplot of *PPARA* expression. **(F)** Focused KEGG GSEA dotplot of representative AMPK-related and metabolism-associated pathways.

#### 3.3.2 Analysis across histopathological severity categories in GSE130970

GSE130970 was further analyzed to examine whether AMPK-associated transcriptional features and selected injury-related genes differed across histopathological severity categories. The AMPK-associated signature differed across NAS, ballooning, steatosis, and lobular inflammation categories, with generally lower scores in the more severe groups (Figures 4A–D). *TGFB1* expression differed across fibrosis stages, with higher expression in more advanced fibrosis categories (Figure 4E). Similarly, *TLR4* and *NFKB2* expression differed across lobular inflammation grades, with higher expression in the more severe categories (Figures 4F,G). These cross-sectional findings indicate that greater histopathological severity is accompanied by lower AMPK-associated transcriptional features and higher expression of selected injury-related genes.

**FIGURE 4:**
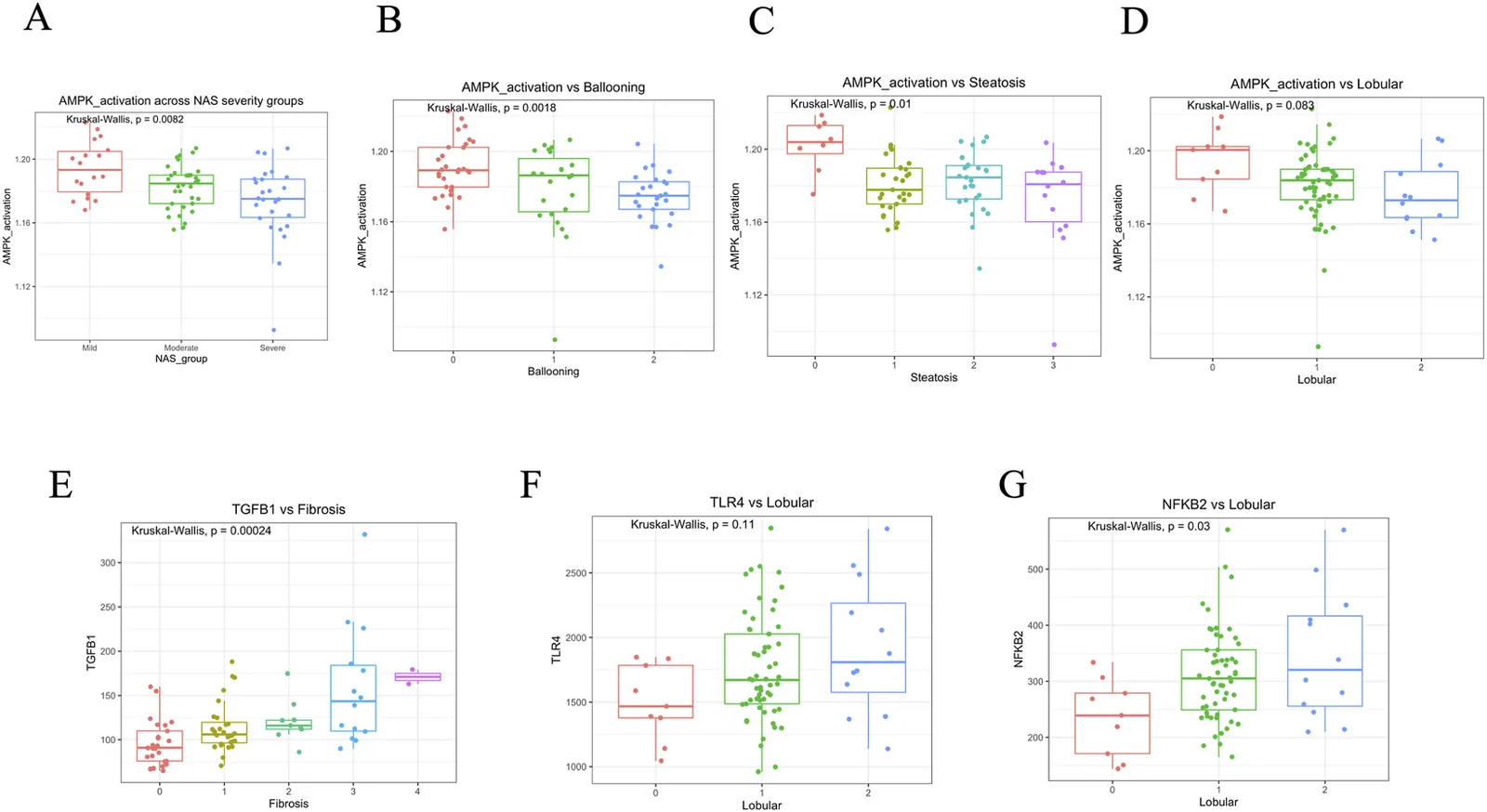
AMPK-associated transcriptional features and selected injury-related genes across histopathological severity categories in GSE130970. **(A–D)** Comparisons of the AMPK activation-associated signature across histopathological severity groups, including NAS, ballooning, steatosis, and lobular inflammation. **(E)** TGFB1 expression across fibrosis stages. **(F,G)** TLR4 and NFKB2 expression across lobular inflammation grades. Kruskal–Wallis tests were used for all analyses.

#### 3.3.3 Integrative analysis of HF919-predicted targets and disease-related transcriptomic alterations identifies disease-relevant functional networks

To examine the disease relevance of the pharmacologically predicted HF919 targets, the predicted target set was integrated with disease-related DEGs derived from GSE126848. Intersection of HF919-predicted targets with NAFLD- and NASH-related DEGs yielded 82 shared genes (Figure 5A). Enrichment analysis indicated that these genes were associated with lipid catabolic regulation, nutrient and ketone responses, PI3K–Akt signaling, injury-related pathways, and efferocytosis-associated processes (Figure 5B).

**FIGURE 5:**
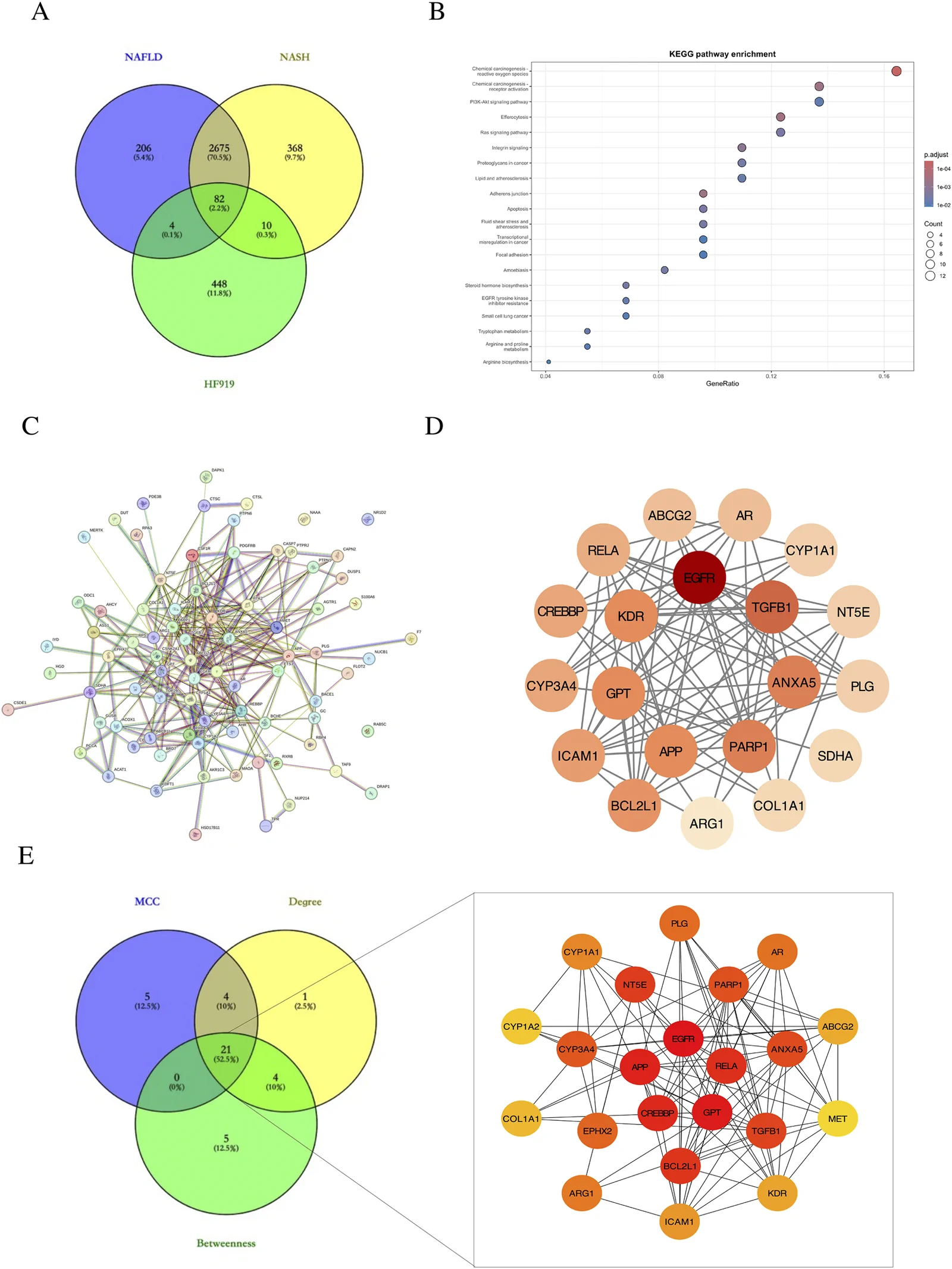
Integrative analysis of HF919-predicted targets and disease-related transcriptomic alterations identifies disease-relevant functional networks in NAFLD. **(A)** Venn diagram showing the overlap among HF919-predicted targets, NAFLD-related DEGs, and NASH-related DEGs from GSE126848. **(B)** KEGG enrichment analysis of the 82 shared genes. **(C)** STRING-based PPI network of the shared genes. **(D)** Top 20 hub-gene subnetwork identified by CentiScaPe analysis. **(E)** Core-gene subnetwork based on the overlapping hub genes identified by MCC, Degree, and Betweenness in CytoHubba.

STRING analysis showed extensive predicted protein–protein interactions among the proteins encoded by the shared genes (Figure 5C). Topological analysis identified highly connected hub nodes, including EGFR, RELA, TGFB1, APP, PARP1, GPT, KDR, BCL2L1, ICAM1, and ARG1, while comparison of MCC, Degree, and Betweenness rankings identified an overlapping subset of hub genes (Figures 5D,E).

These integrative analyses identified disease-relevant overlap between HF919-predicted targets and transcriptional alterations in human NAFLD/NASH liver. The results were used to prioritize metabolic and injury-related candidate pathways for subsequent experimental evaluation and were not interpreted as evidence that HF919 directly regulates these genes in human liver tissue.

### 3.4 Molecular docking predicts potential ligand–target compatibility

To computationally assess potential ligand–target compatibility, the five selected candidate HF919 compounds were docked with the AMPK, TGFB1, and ARG1. All ligand–receptor pairs yielded AutoDock Vina docking scores below −5 kcal/mol (Figure 6B). The lowest-scoring pose for each ligand–receptor pair was selected for visualization, and representative predicted docking poses are shown in Figure 6A. These results indicate potential computational compatibility between the selected compounds and the receptor structures but do not establish physical binding or functional target engagement.

**FIGURE 6:**
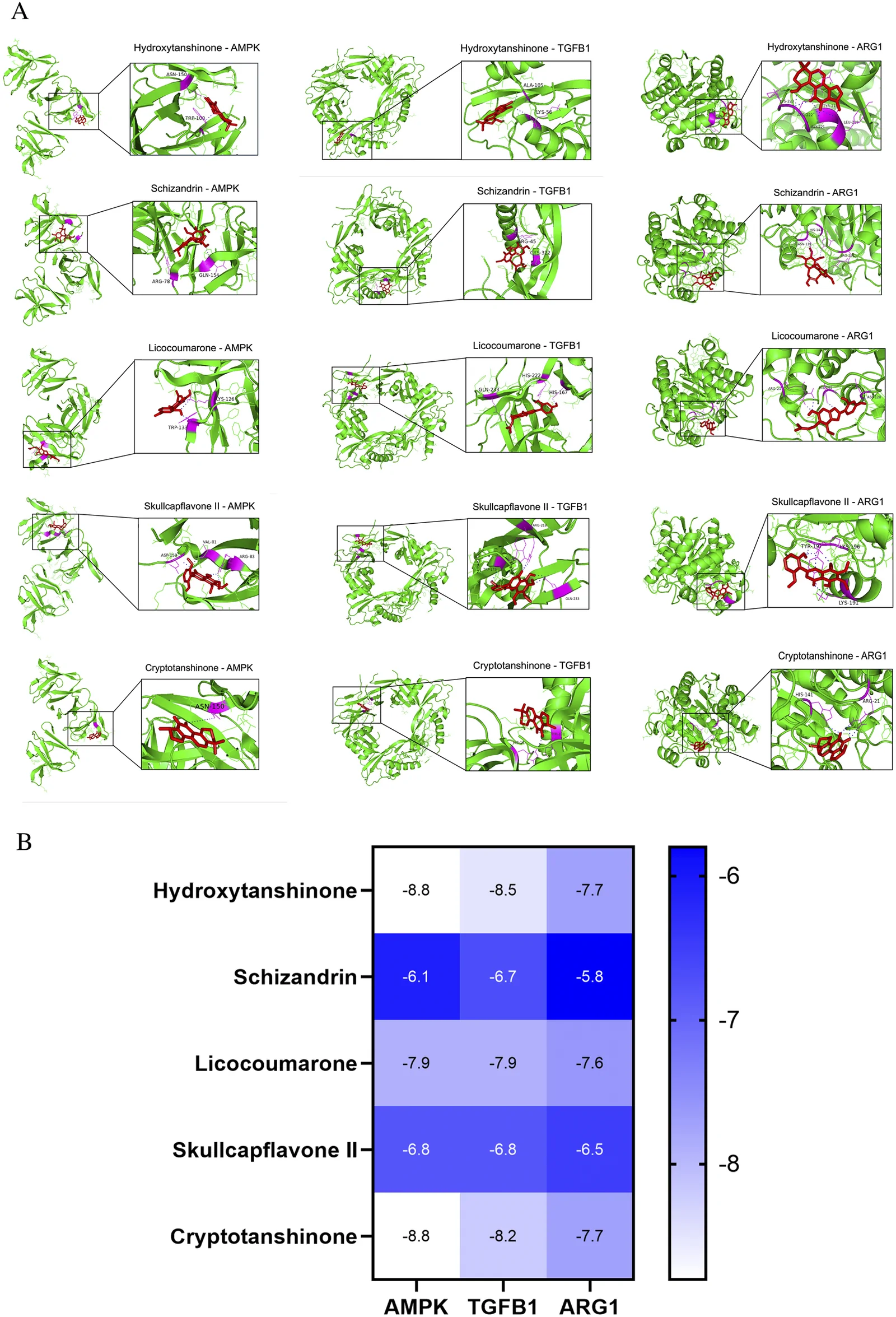
Molecular docking of selected HF919 compounds with representative targets. **(A)** Predicted docking poses of five selected HF919 compounds with the AMPK, TGFB1, and ARG1. **(B)** AutoDock Vina docking scores (kcal/mol) for the corresponding ligand–target pairs.

### 3.5 HF919 improves hepatic steatosis and biochemical abnormalities in NAFLD rats

Gross liver examination showed that livers from the NAFLD group were enlarged, yellowish, and blunt-edged, consistent with fatty infiltration, whereas HF919 treatment visibly ameliorated these morphological abnormalities (Figures 7A–C). Quantitative analysis further showed that the liver-to-body weight ratio was significantly increased in the NAFLD group and was markedly reduced after HF919 treatment (Figure 7D).

**FIGURE 7:**
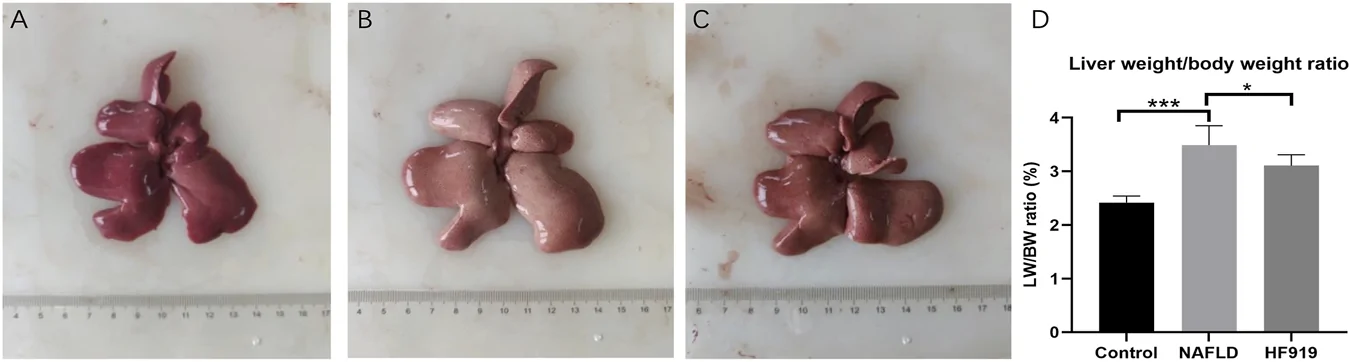
Effects of HF919 on gross liver appearance and liver-to-body weight ratio in NAFLD rats. **(A–C)** Representative gross liver appearance of the Control, NAFLD, and HF919-treated groups. **(D)** Quantitative analysis of the liver-to-body weight ratio (LW/BW, %), calculated as liver weight/body weight × 100%. Data are presented as mean ± SD (n = 6 per group). *P < 0.05, ***P < 0.001 compared with the NAFLD group.

Histopathological examination by H&E staining demonstrated prominent hepatocellular steatosis and disorganization of hepatic architecture in the NAFLD group, while HF919 treatment markedly alleviated these pathological changes (Figure 8A). Oil Red O staining further demonstrated prominent hepatic lipid accumulation in NAFLD rats. ImageJ-based quantification showed that the Oil Red O-positive area was significantly increased in the NAFLD group compared with the Control group (*P* = 0.0008) and significantly reduced after HF919 treatment compared with the NAFLD group (*P* = 0.0089) (Figure 8B; Table 3).

**FIGURE 8:**
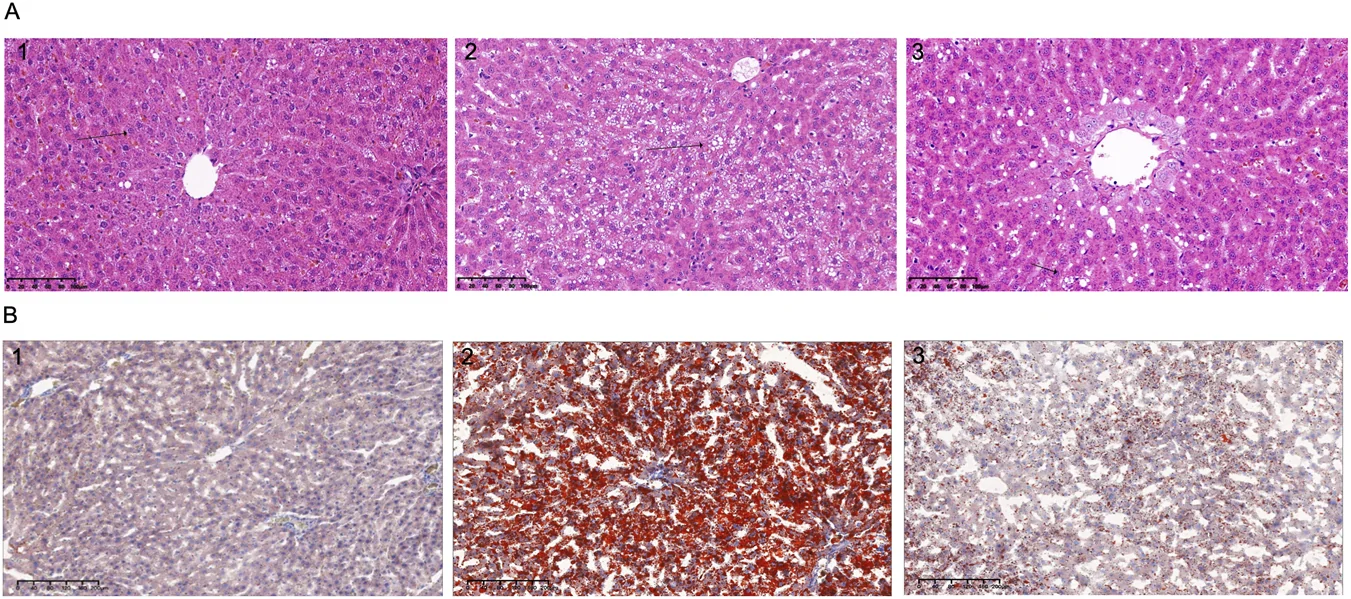
Effects of HF919 on Hepatic Histopathology in NAFLD Rats. **(A)** Representative H&E-stained liver sections (original magnification, ×200). **(B)** Representative Oil Red O-stained liver sections showing hepatic lipid accumulation (original magnification, ×100). In both panels, the groups are: (1) Control, (2) NAFLD, and (3) HF919 treatment. One representative image is shown per group (n = 3 per group).

**TABLE 3:** Quantitative analysis of hepatic Oil Red O-stained lipid droplets in rats.

Slice	Count	Total area (pixel^2^)	Average size (pixel^2^)	%Area
Control	6827 ± 3533	85,973 ± 32,238	13.324 ± 2.498	3.447 ± 1.292
NAFLD	3131 ± 664	1363138 ± 97,406	455.526 ± 143.091	54.647 ± 3.905
HF919	9629 ± 3200	369,979 ± 150,247	37.868 ± 7.720	17.946 ± 8.726

Data are presented as mean ± SD (n = 3 rats per group).

Serum biochemical analysis showed that, compared with Control rats, NAFLD rats had significantly higher TG, TC, LDL-C, FFA, ALT, and AST levels and lower HDL-C levels (Figure 9A). Compared with the NAFLD group, HF919 treatment significantly reduced serum TG, TC, LDL-C, FFA, ALT, and AST levels, whereas no significant difference in HDL-C was observed between the NAFLD and HF919 groups. Hepatic TG and TC contents were also significantly increased in the NAFLD group and reduced after HF919 treatment (Figure 9B). These findings demonstrate that HF919 alleviated hepatic lipid accumulation and improved several lipid-related and liver injury-associated biochemical abnormalities in HFD-induced NAFLD rats.

**FIGURE 9:**
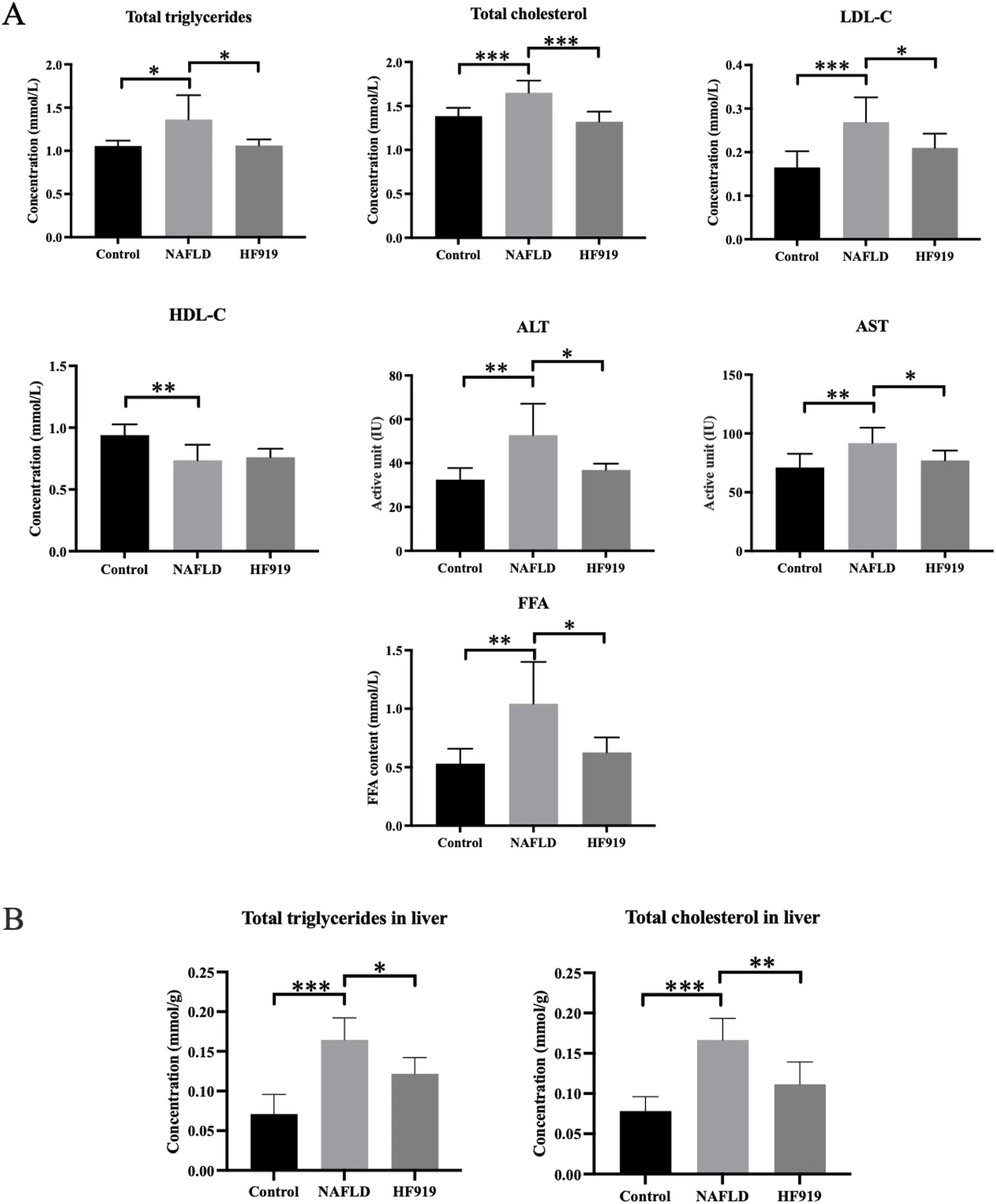
Effects of HF919 on serum biochemical parameters and hepatic lipid contents in NAFLD rats. **(A)** Serum biochemical parameters (n = 8 per group). **(B)** Hepatic lipid markers (n = 6 per group). Data are presented as mean ± SD. *P < 0.05, **P < 0.01, ***P < 0.001 compared with the NAFLD group.

### 3.6 HF919 treatment is accompanied by increased hepatic p-AMPK and changes in selected metabolic and injury-related markers

qRT-PCR analysis showed that HF919 significantly reduced the hepatic mRNA expression of *Tlr4, Nfkb2,* and *Tgfb1* and increased the expression of *Ppara, Arg1,* and *Mertk* compared with the NAFLD group (Figure 10A). Thus, HF919 treatment was accompanied by changes in selected lipid metabolism-, injury/inflammation-, fibrosis-, and macrophage-associated transcripts.

**FIGURE 10:**
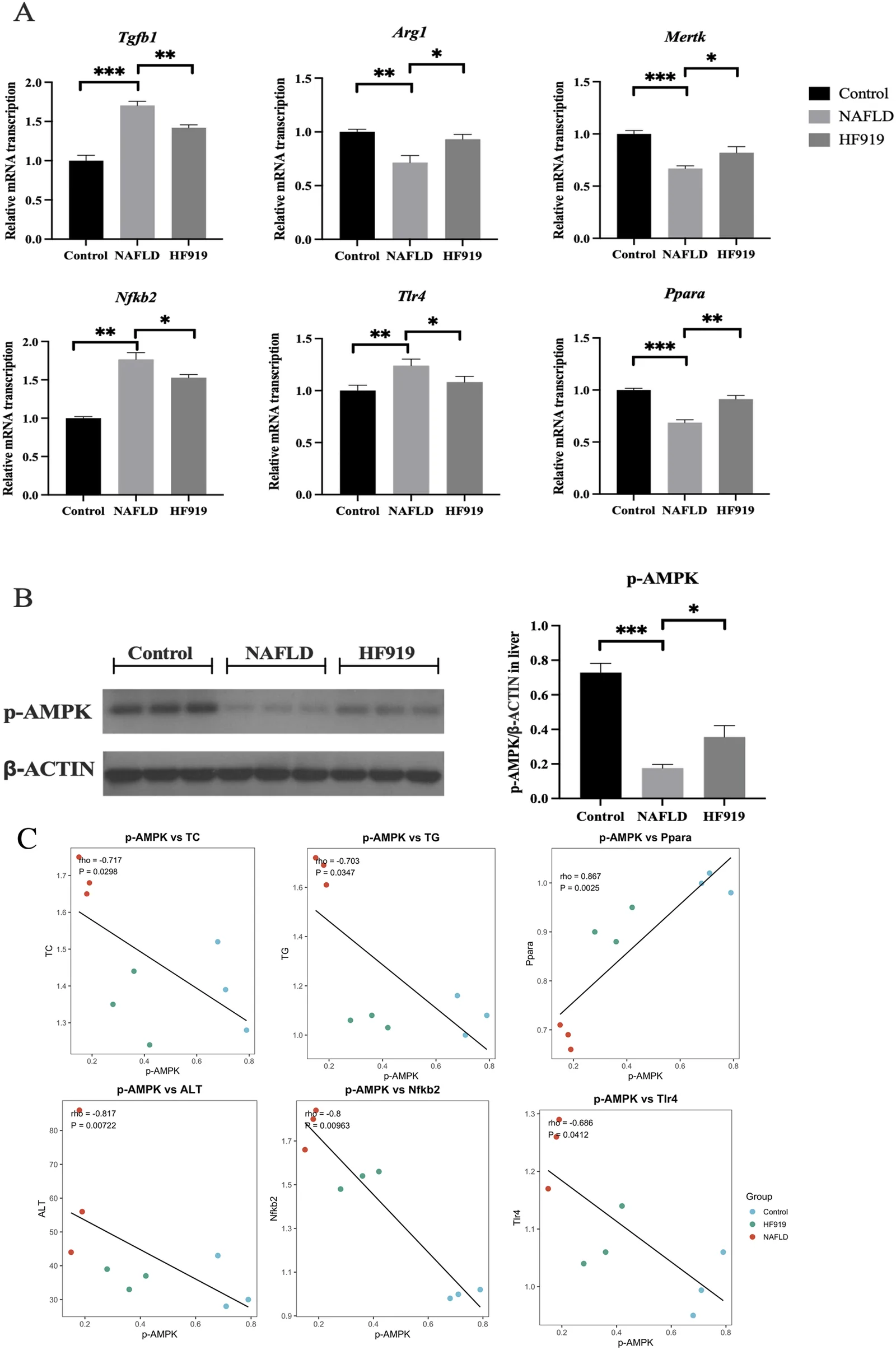
HF919 alters selected hepatic metabolic, injury-related, and macrophage-associated markers in NAFLD rats. **(A)** Hepatic mRNA expression of *Ppara*, *Tlr4*, *Nfkb2*, *Tgfb1*, *Mertk*, and *Arg1* measured by qRT-PCR. **(B)** Representative Western blot and densitometric analysis of hepatic p-AMPK abundance. Data in **(A,B)** are presented as mean ± SD (n = 3 per group). **(C)** Exploratory Spearman correlation analysis between hepatic p-AMPK and selected metabolic and injury-related parameters (n = 9 in total; 3 per group). *P < 0.05, **P < 0.01, ***P < 0.001 compared with the NAFLD group.

Western blot analysis further showed significantly increased hepatic p-AMPK abundance in the HF919 group compared with the NAFLD group (Figure 10B). Together with increased *Ppara* expression and improved lipid-related parameters, this finding is consistent with the possible involvement of AMPK-associated regulation in the response to HF919.

To further examine the relationship between hepatic p-AMPK abundance and selected metabolic and injury-related parameters, exploratory Spearman correlation analyses were performed using matched individual animals (Figure 10C). Hepatic p-AMPK abundance was negatively correlated with serum TG, TC, and ALT, positively correlated with *Ppara* expression, and negatively correlated with *Tlr4* and *Nfkb2* expression. These exploratory analyses indicate associations between higher hepatic p-AMPK abundance and more favorable metabolic and selected gene-expression profiles across the analyzed animals but do not establish a causal relationship.

### 3.7 HF919 reduces hepatic F4/80-positive staining in NAFLD rats

Immunohistochemical analysis showed sparse F4/80-positive staining in the Control group, whereas staining was markedly increased in the NAFLD group (Figure 11). ImageJ-based quantification confirmed a significantly higher F4/80-positive area in the NAFLD group than in the Control group (*P* = 0.0093), which was significantly reduced by HF919 treatment (*P* = 0.0032) (Table 4). These findings indicate reduced hepatic F4/80-positive macrophage-associated staining after HF919 treatment. Because macrophage polarization and functional activity were not directly assessed, no specific macrophage phenotype or function was inferred.

**FIGURE 11:**
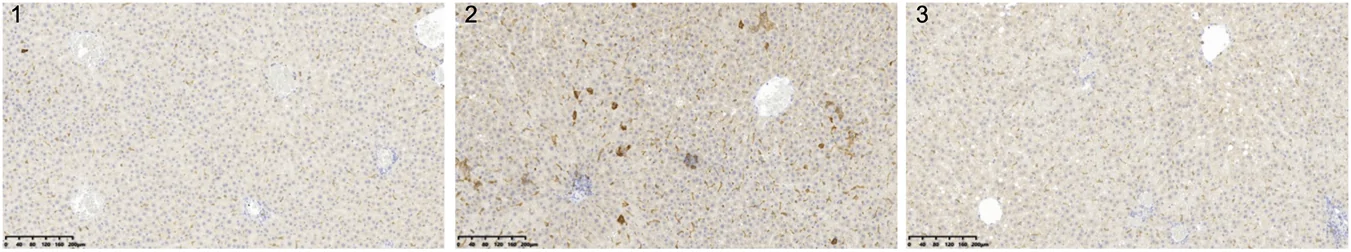
Effects of HF919 on hepatic F4/80-positive staining in NAFLD rats. Representative F4/80 immunohistochemical staining of liver sections from the (1) Control, (2) NAFLD, and (3) HF919 groups (original magnification, ×200). F4/80-positive staining is shown as brown signals. One representative image is shown per group (n = 3 per group).

**TABLE 4:** Quantitative analysis of hepatic F4/80-positive staining.

Slice	Count	Total area (pixel^2^)	Average size (pixel^2^)	%Area
Control	886.67 ± 138.08	27,954.67 ± 4308.02	32.051 ± 7.360	1.387 ± 0.176
NAFLD	2220.67 ± 831.39	126,422.67 ± 21,874.15	65.889 ± 38.448	6.247 ± 0.903
HF919	925.00 ± 99.53	35,015.67 ± 16,740.90	36.856 ± 14.747	1.740 ± 0.839

Data are presented as mean ± SD (n = 3 rats per group).

### 3.8 Exploratory analysis of fecal SCFA profiles following HF919 treatment

Targeted analysis identified differences in fecal SCFA levels among the experimental groups (Figure 12A). Compared with the Control group, the NAFLD group showed higher levels of acetic acid, butyric acid, isobutyric acid, and isovaleric acid and lower levels of valeric acid and isocaproic acid. HF919 treatment shifted several of these SCFAs toward the levels observed in the Control group, indicating a partial shift of the fecal SCFA profile toward the control-associated pattern. Hierarchical clustering further showed that the overall SCFA profile of the HF919-treated group was more similar to that of the Control group than to that of the untreated NAFLD group (Figure 12B).

**FIGURE 12:**
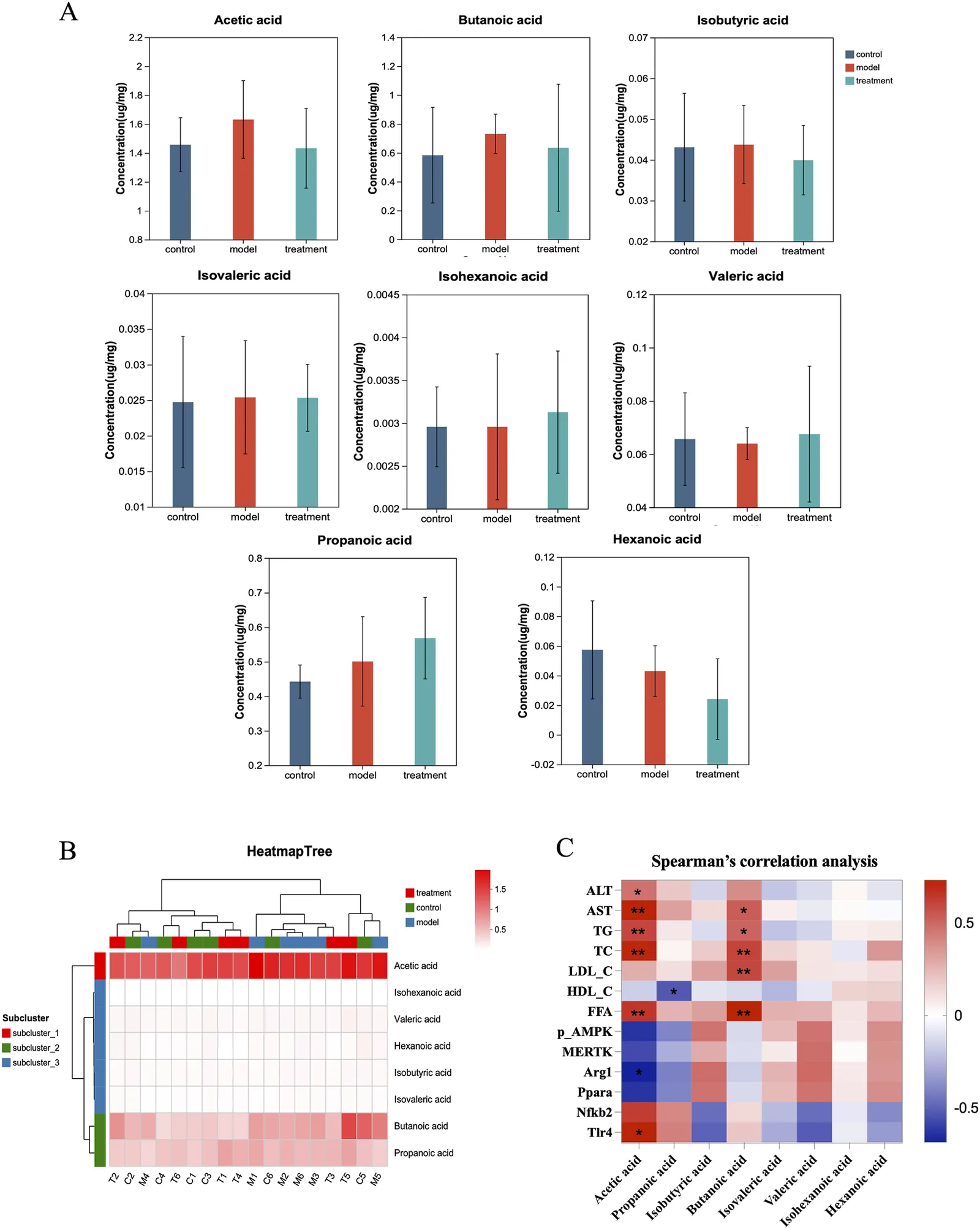
Fecal SCFA profiles and exploratory correlations with NAFLD-related parameters following HF919 treatment. **(A)** Differential abundance analysis of fecal SCFAs (n = 6 per group). **(B)** Hierarchical clustering analysis of SCFA profiles. C, Control group; M/Model, NAFLD group; T/Treatment, HF919 group. **(C)** Spearman correlation heatmap showing the relationships between fecal SCFAs and NAFLD-related parameters, including biochemical indicators, hepatic lipid indices, and selected hepatic gene/protein markers. Correlations were calculated using available matched data from individual animals. Red indicates a positive correlation (*r* > 0) and blue indicates a negative correlation (*r* < 0); color intensity reflects correlation strength. *P < 0.05, **P < 0.01.

Exploratory Spearman correlation analysis was performed to examine the relationships between fecal SCFAs and selected NAFLD-related biochemical and hepatic parameters using available matched individual-animal data (Figure 12C). Higher acetic acid and butyric acid levels were positively correlated with serum ALT and AST, lipid-related indices including TG, TC, and FFA, and the expression of *Nfkb2* and *Tgfb1*. These SCFAs were negatively correlated with hepatic p-AMPK abundance and with the macrophage-associated genes *Mertk* and *Arg1*. Valeric acid showed an opposite correlation pattern for several of these parameters. Taken together, the group-wise shifts in SCFA levels and the observed correlation patterns suggest that HF919 treatment was associated with remodeling of the fecal SCFA profile in parallel with improvements in hepatic metabolic and injury-related parameters. These findings support SCFA-related metabolic changes as a potential accompanying component of the response to HF919, although they do not establish that SCFA alterations mediated the hepatic effects of HF919 or demonstrate a causal gut–liver mechanism.

## 4 Discussion

NAFLD is characterized by hepatic lipid accumulation, metabolic dysregulation, and progressive liver injury. In the present study, UPLC-HRMS, network pharmacology, public liver transcriptomic analyses, molecular docking, and an HFD-induced rat model were integrated to examine the effects of HF919 and the possible involvement of AMPK-associated regulation. HF919 alleviated hepatic steatosis and improved lipid-related and liver injury-associated biochemical abnormalities. These effects were accompanied by increased hepatic p-AMPK abundance and *Ppara* expression, lower expression of selected injury-related genes, changes in macrophage-associated markers, and shifts in fecal SCFA profiles. The combined findings are consistent with possible involvement of AMPK-associated metabolic regulation, whereas the macrophage- and SCFA-related findings represent secondary and exploratory observations. A schematic summary of the main findings and proposed associations is shown in Figure 13.

**FIGURE 13:**
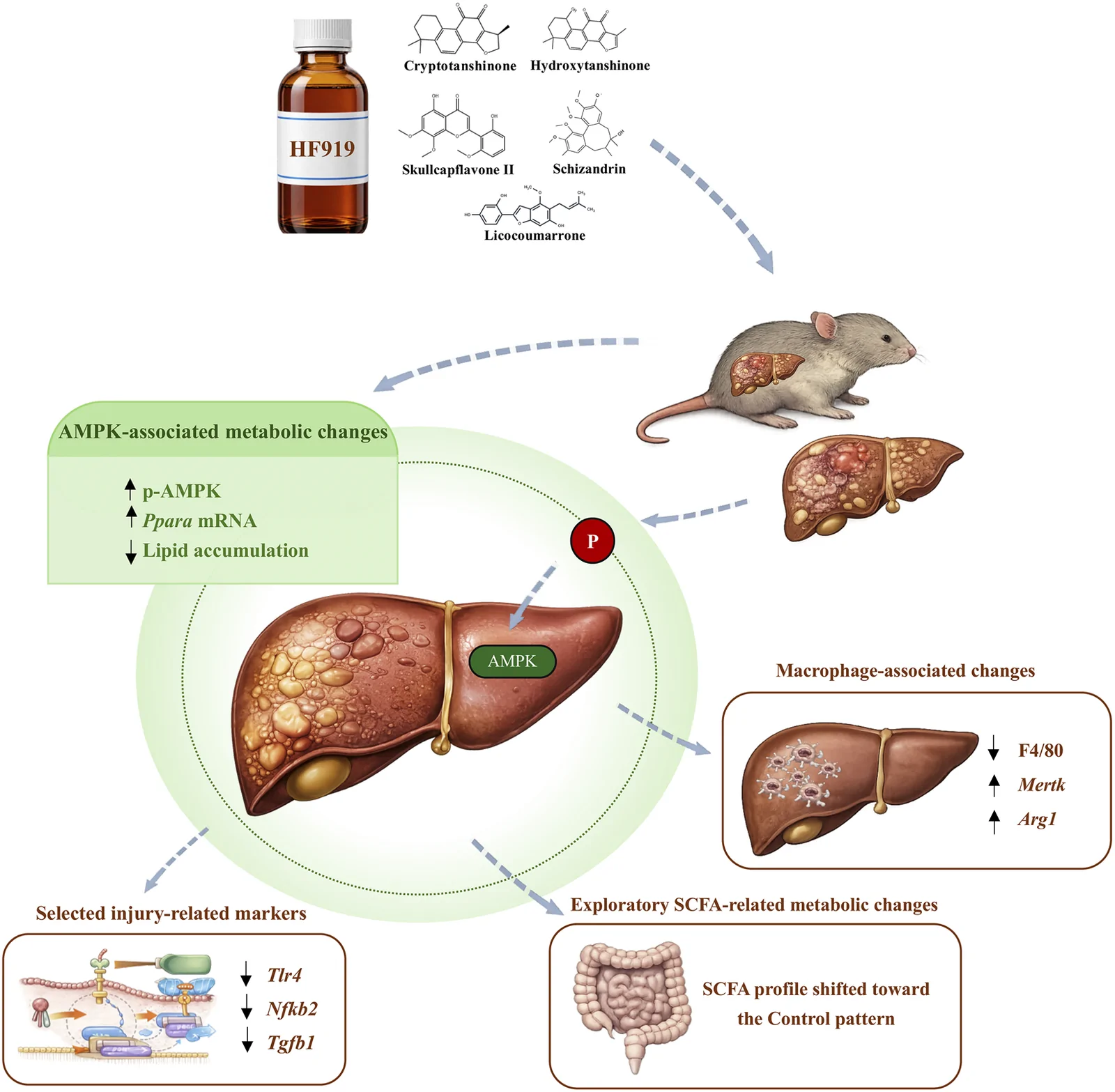
Schematic summary of the main findings and proposed associations related to HF919 treatment in NAFLD. HF919 ameliorated hepatic steatosis and metabolic abnormalities in HFD-induced NAFLD rats. These effects were accompanied by increased hepatic p-AMPK abundance and *Ppara* expression, lower expression of selected injury- and fibrosis-associated genes, reduced F4/80-positive staining, changes in macrophage-associated gene expression, and exploratory shifts in fecal SCFA profiles. The proposed associations shown in the schematic do not imply established causal relationships.

A major pathological feature of NAFLD is the imbalance among lipid synthesis, lipid utilization, and fatty acid oxidation (Duell et al., 2022; Luukkonen et al., 2022). AMPK is a key energy sensor that regulates these processes by promoting fatty acid oxidation and restraining lipogenic processes under metabolic stress (Steinberg and Hardie, 2023). In the present study, HF919 treatment increased hepatic p-AMPK abundance and *Ppara* expression while reducing hepatic steatosis and improving serum and hepatic lipid indices, consistent with the possible involvement of AMPK-associated metabolic regulation. Public transcriptomic datasets provided additional disease-context support. In GSE126848, NASH liver samples showed lower AMPK-related and FAO-protective scores together with lower *PPARA* expression, whereas GSE130970 showed generally lower AMPK-associated signature scores in more severe histopathological categories. Although these human datasets do not provide evidence of a response to HF919, they support the disease relevance of AMPK- and fatty-acid-oxidation-related transcriptional dysregulation in NAFLD. Together, these findings support the possible involvement of AMPK-associated regulation in the metabolic effects observed following HF919 treatment.

HF919 treatment was also accompanied by changes in selected injury-related genes. Hepatic mRNA expression of *Tlr4, Nfkb2,* and *Tgfb1* was lower after HF919 treatment than in the untreated NAFLD group. These genes are associated with inflammatory responses, tissue injury, and fibrogenic processes that contribute to NAFLD pathogenesis (Sun et al., 2023; Luedde and Schwabe, 2011; Fabregat et al., 2016). In the public transcriptomic analysis, their expression also differed across relevant histopathological severity categories, supporting their disease relevance. However, because the present study assessed selected transcript levels rather than pathway activity or fibrotic outcomes, these findings are best interpreted as changes in injury- and fibrosis-associated gene expression rather than direct evidence of suppression of inflammatory or fibrogenic signaling. Exploratory Spearman analysis further showed that higher hepatic p-AMPK abundance was associated with more favorable metabolic indices and selected gene-expression profiles, although these correlations do not establish a causal relationship.

Macrophage-associated changes represented a secondary but relevant observation in the present study. During NASH development, monocyte-derived macrophages accumulate and form distinct lipid-associated populations that participate in hepatic tissue remodeling (Daemen et al., 2021). Hepatic F4/80-positive staining was significantly increased in the NAFLD group and significantly reduced after HF919 treatment, while hepatic expression of the macrophage-associated genes *Mertk* and *Arg1* was increased. These findings indicate changes in hepatic F4/80-positive staining and macrophage-associated gene expression following HF919 treatment. However, F4/80 staining together with *Mertk* and *Arg1* expression does not establish macrophage polarization, reparative function, or enhanced efferocytosis.

Fecal SCFA profiling provided an additional exploratory metabolic observation. SCFAs have been implicated in metabolic regulation and the pathophysiology of NAFLD/MASLD (Li et al., 2024; Zhang et al., 2025). In the present study, several SCFAs shifted toward the levels observed in the Control group following HF919 treatment, and hierarchical clustering showed greater similarity between the overall SCFA profiles of the HF919 and Control groups than between the HF919 and untreated NAFLD groups. Exploratory correlations further linked individual SCFAs with hepatic lipid indices, p-AMPK abundance, and selected injury- and macrophage-associated genes. Together, these observations suggest that SCFA-related metabolic changes may accompany the hepatic response to HF919. However, the present data do not establish that SCFA alterations mediate the hepatic effects of HF919 or demonstrate a causal gut–liver mechanism.

An important strength of this study is the integration of complementary levels of evidence. Network pharmacology identified candidate metabolic and injury-related pathways, while public human liver transcriptomic datasets provided independent disease-context evidence for AMPK- and fatty-acid-oxidation-related transcriptional dysregulation in NAFLD. Molecular docking yielded favorable predicted docking scores between selected candidate HF919 compounds and representative receptor structures, whereas the *in vivo* experiments directly demonstrated reduced hepatic steatosis and biochemical abnormalities together with increased hepatic p-AMPK abundance and changes in selected hepatic markers following HF919 treatment.

Several limitations should be acknowledged. First, although increased hepatic p-AMPK abundance accompanied the beneficial effects of HF919, AMPK inhibition or pathway-blocking experiments were not performed; therefore, the present study does not establish a causal requirement for AMPK signaling in these effects. Second, only a limited set of lipid metabolism-related molecular markers was assessed, and fatty-acid uptake, *de novo* lipogenesis, triglyceride synthesis, and lipolysis were not directly characterized. Third, no pharmacological comparator was included, precluding conclusions regarding the relative efficacy, superiority, or equivalence of HF919 compared with established pharmacological interventions. Fourth, reduced hepatic F4/80-positive staining and changes in *Mertk* and *Arg1* expression do not establish macrophage polarization, reparative function, or efferocytic activity. Fifth, fecal SCFA profiling was exploratory, and the observed associations between SCFAs and hepatic parameters do not establish that SCFA alterations mediated the effects of HF919 or demonstrate a causal gut–liver mechanism. In addition, reduced *Tgfb1* expression alone does not establish an anti-fibrotic effect in the absence of dedicated histological or molecular assessment of fibrosis. Finally, because HF919 is a multi-component formulation, the relative contributions of individual candidate constituents remain to be clarified.

Taken together, HF919 ameliorated hepatic steatosis and metabolic abnormalities in HFD-induced NAFLD rats. These effects were accompanied by increased hepatic p-AMPK abundance and changes in selected lipid metabolism-related markers, supporting the possible involvement of AMPK-associated metabolic regulation. HF919 treatment was also accompanied by changes in selected injury-related genes, reduced hepatic F4/80-positive staining, macrophage-associated gene-expression changes, and exploratory shifts in fecal SCFA profiles. These secondary findings broaden the biological context of the metabolic response to HF919 but do not establish additional causal mechanisms. Further mechanistic and comparative studies are required to define the contribution of AMPK signaling and to clarify the biological significance of the macrophage- and SCFA-associated changes.

## 5 Conclusion

In summary, HF919 ameliorated hepatic steatosis and metabolic abnormalities in HFD-induced NAFLD rats. The accompanying increase in hepatic p-AMPK abundance and *Ppara* expression, together with disease-context evidence from human liver transcriptomic datasets, supports the possible involvement of AMPK-associated metabolic regulation. Changes in selected injury-related and macrophage-associated markers and fecal SCFA profiles provided additional, mainly secondary or exploratory observations. Further mechanistic studies are needed to establish the causal contribution of AMPK signaling to the effects of HF919.

## Data Availability

Publicly available datasets were analyzed in this study. This data can be found here: https://www.ncbi.nlm.nih.gov/geo/query/acc.cgi?acc=GSE126848 and https://www.ncbi.nlm.nih.gov/geo/query/acc.cgi?acc=GSE130970

## References

[B1] Chen M. Xing J. Pan D. Peng X. Gao P. (2020). Chinese herbal medicine mixture 919 syrup alleviates nonalcoholic fatty liver disease in rats by inhibiting the NF-κB pathway. Biomed. Pharmacother. 128, 110286. 10.1016/j.biopha.2020.110286 32521450

[B2] Chen M. Xing J. Pan D. Gao P. (2021). Effect of Chinese herbal medicine mixture 919 syrup on regulation of the ghrelin pathway and intestinal microbiota in rats with non-alcoholic fatty liver disease. Front. Microbiol. 12, 793854. 10.3389/fmicb.2021.793854 35003024 PMC8740226

[B3] Cusi K. Alkhouri N. Harrison S. A. Fouqueray P. Moller D. E. Hallakou-Bozec S. (2021). Efficacy and safety of PXL770, a direct AMP kinase activator, for the treatment of non-alcoholic fatty liver disease (STAMP-NAFLD): a randomised, double-blind, placebo-controlled, phase 2a study. Lancet Gastroenterol. Hepatol. 6 (11), 889–902. 10.1016/S2468-1253(21)00300-9 34560015

[B4] Daemen S. Gainullina A. Kalugotla G. He L. Chan M. M. Beals J. W. (2021). Dynamic shifts in the composition of resident and recruited macrophages influence tissue remodeling in NASH. Cell Rep. 34 (2), 108626. 10.1016/j.celrep.2020.108626 33440159 PMC7877246

[B5] Ding Y. Yanagi K. Cheng C. Alaniz R. C. Lee K. Jayaraman A. (2019). Interactions between gut microbiota and non-alcoholic liver disease: the role of microbiota-derived metabolites. Pharmacol. Res. 141, 521–529. 10.1016/j.phrs.2019.01.029 30660825 PMC6392453

[B6] Duell P. B. Welty F. K. Miller M. Chait A. Hammond G. Ahmad Z. (2022). Nonalcoholic fatty liver disease and cardiovascular risk: a scientific statement from the American heart association. Arterioscler. Thromb. Vasc. Biol. 42 (6), e168–e185. 10.1161/ATV.0000000000000153 35418240

[B7] Fabregat I. Moreno-Caceres J. Sanchez A. Dooley S. Dewidar B. Giannelli G. (2016). TGF-β signalling and liver disease. FEBS J. 283 (12), 2219–2232. 10.1111/febs.13665 26807763

[B8] Friedman S. L. Neuschwander-Tetri B. A. Rinella M. Sanyal A. J. (2018). Mechanisms of NAFLD development and therapeutic strategies. Nat. Med. 24 (7), 908–922. 10.1038/s41591-018-0104-9 29967350 PMC6553468

[B9] Gao P. Zhang Y. (2005). Experimental study on 919 syrup in protecting liver. Shanghai J. Tradit. Chin. Med. 39 (5), 53–54. 10.3969/j.issn.1007-1334.2005.05.024

[B10] Garcia D. Hellberg K. Chaix A. Wallace M. Herzig S. Badur M. G. (2019). Genetic liver-specific AMPK activation protects against diet-induced obesity and NAFLD. Cell Rep. 26 (1), 192–208.e6. 10.1016/j.celrep.2018.12.036 PMC6344045 30605676

[B11] Horai H. Arita M. Kanaya S. Nihei Y. Ikeda T. Suwa K. (2010). MassBank: a public repository for sharing mass spectral data for life sciences. J. Mass Spectrom. 45 (7), 703–714. 10.1002/jms.1777 20623627

[B12] Huang J. Gao P. (2002). Experimental study on anti-duck hepatitis B virus with 919 syrup. Chin. J. Integr. Tradit. West. Med. Liver Dis. 12 (1), 28–30. 10.3969/j.issn.1005-0264.2002.01.012

[B13] Huang J. Huang H. Liu A. (1995). Clinical study of 919 syrup in the treatment of chronic hepatitis B. China J. Tradit. Chin. Med. Pharm. 10 (1), 17–18. 10.3321/j.issn:1673-1727.1995.01.018

[B14] Huang J. Wu Z. Liu A. (2003). Experimental study of 919 syrup anti-hepatic fibrosis in rats. Chin. J. Integr. Tradit. West. Med. Liver Dis. 13 (3), 160–162. 10.3969/j.issn.1005-0264.2003.03.012

[B15] Li X. He M. Yi X. Lu X. Zhu M. Xue M. (2024). Short-chain fatty acids in nonalcoholic fatty liver disease: new prospects for short-chain fatty acids as therapeutic targets. Heliyon 10 (5), e26991. 10.1016/j.heliyon.2024.e26991 38486722 PMC10937592

[B16] Li C. Yao J. Yang C. Yu S. Yang Z. Wang L. (2025). Gut microbiota-derived short-chain fatty acids act as mediators of the gut-liver-brain axis. Metab. Brain Dis. 40 (2), 122. 10.1007/s11011-025-01554-5 39921774

[B17] Luedde T. Schwabe R. F. (2011). NF-κB in the liver—linking injury, fibrosis and hepatocellular carcinoma. Nat. Rev. Gastroenterol. Hepatol. 8 (2), 108–118. 10.1038/nrgastro.2010.213 21293511 PMC3295539

[B18] Luukkonen P. K. Qadri S. Ahlholm N. Porthan K. Mannisto V. Sammalkorpi H. (2022). Distinct contributions of metabolic dysfunction and genetic risk factors in the pathogenesis of non-alcoholic fatty liver disease. J. Hepatol. 76 (3), 526–535. 10.1016/j.jhep.2021.10.013 PMC8852745 34710482

[B19] Powell E. E. Wong V. W. S. Rinella M. (2021). Non-alcoholic fatty liver disease. Lancet 397 (10290), 2212–2224. 10.1016/S0140-6736(20)32511-3 33894145

[B20] Shashni B. Tajika Y. Ikeda Y. Nishikawa Y. Nagasaki Y. (2023). Self-assembling polymer-based short chain fatty acid prodrugs ameliorate non-alcoholic steatohepatitis and liver fibrosis. Biomaterials 295, 122047. 10.1016/j.biomaterials.2023.122047 36840994

[B21] Smith C. A. O'Maille G. Want E. J. Qin C. Trauger S. A. Brandon T. R. (2005). METLIN: a metabolite mass spectral database. Ther. Drug Monit. 27 (6), 747–751. 10.1097/01.ftd.0000179845.53213.39 16404815

[B22] Smith B. K. Marcinko K. Desjardins E. M. Lally J. S. Ford R. J. Steinberg G. R. (2016). Treatment of nonalcoholic fatty liver disease: role of AMPK. Am. J. Physiol. Endocrinol. Metab. 311 (4), E730–E740. 10.1152/ajpendo.00225.2016 27577854

[B23] Steinberg G. R. Hardie D. G. (2023). New insights into activation and function of the AMPK. Nat. Rev. Mol. Cell Biol. 24 (4), 255–272. 10.1038/s41580-022-00547-x 36316383

[B24] Sun B. L. Sun X. Kempf C. L. Song J. H. Casanova N. G. Camp S. M. (2023). Involvement of eNAMPT/TLR4 inflammatory signaling in progression of non-alcoholic fatty liver disease, steatohepatitis, and fibrosis. FASEB J. 37 (3), e22825. 10.1096/fj.202201972RR 36809677 PMC11265521

[B25] Wang S. Xu H. Du R. L. Wei C. S. Cui X. Zhang C. M. (2025). Underlying mechanisms of traditional Chinese medicine in treating metabolic dysfunction-associated steatohepatitis: evidences from preclinical and clinical studies. J. Ethnopharmacol. 352, 120192. 10.1016/j.jep.2025.120192 40582580

[B26] Williams C. D. Stengel J. Asike M. I. Torres D. M. Shaw J. Contreras M. (2011). Prevalence of nonalcoholic fatty liver disease and nonalcoholic steatohepatitis among a largely middle-aged population utilizing ultrasound and liver biopsy: a prospective study. Gastroenterology 140 (1), 124–131. 10.1053/j.gastro.2010.09.038 20858492

[B27] Wishart D. S. Guo A. Oler E. Wang F. Anjum A. Peters H. (2022). HMDB 5.0: the human metabolome database for 2022. Nucleic Acids Res. 50 (D1), D622–D631. 10.1093/nar/gkab1062 34986597 PMC8728138

[B28] Xu Y. M. Xiao Q. Gao Q. Li B. Watanabe K. Chen B. Q. (2017). Effects of the Chinese herbal medicine mixture 919 syrup on the isolation stress response in postpartum mice. Int. J. Clin. Exp. Med. 10 (4), 6527–6537. 10.1166/jbt.2017.1585

[B29] Younossi Z. M. Golabi P. Paik J. M. Henry A. Van Dongen C. Henry L. (2023). The global epidemiology of nonalcoholic fatty liver disease (NAFLD) and nonalcoholic steatohepatitis (NASH): a systematic review. Hepatology 77 (4), 1335–1347. 10.1097/HEP.0000000000000004 36626630 PMC10026948

[B30] Zhang S. Zhao J. Xie F. He H. Johnston L. J. Dai X. (2021). Dietary fiber-derived short-chain fatty acids: a potential therapeutic target to alleviate obesity-related nonalcoholic fatty liver disease. Obes. Rev. 22 (11), e13316. 10.1111/obr.13316 34279051

[B31] Zhang W. Y. Wang M. H. Xie C. (2024). Potential of traditional Chinese medicine in the treatment of nonalcoholic fatty liver disease: a promising future. World J. Gastroenterol. 30 (43), 4597–4601. 10.3748/wjg.v30.i43.4597 39575403 PMC11572638

[B32] Zhang C. Y. Liu S. Sui Y. X. Yang M. (2025). Roles of short-chain fatty acids in metabolic dysfunction-associated steatotic liver disease and metabolic dysfunction-associated steatohepatitis. World J. Hepatol. 17 (11), 113756. 10.4254/wjh.v17.i11.113756 41368119 PMC12683382

[B33] Zhao P. Saltiel A. R. (2020). From overnutrition to liver injury: AMP-activated protein kinase in nonalcoholic fatty liver diseases. J. Biol. Chem. 295 (34), 12279–12289. 10.1074/jbc.REV120.011356 32651233 PMC7443502

