# Supplementary material for: Hepatoprotective Formula 919 ameliorates hepatic steatosis and metabolic disturbance in NAFLD with involvement of AMPK signaling
Source: Front Pharmacol. 2026 Sep 28;17:1892654. doi: 10.3389/fphar.2026.1892654 (PMC13654718; doi:10.3389/fphar.2026.1892654)
Supplement: Supplementary file 1 [file Supplementaryfile1.docx]

A

B

C

D

E

F

**Supplementary Fig. S1. Parallel transcriptomic analysis of GSE126848 supports suppression of AMPK-associated protective metabolic programs in NAFLD liver.** (A) Volcano plot of differentially expressed genes between Control and NAFLD liver samples. (B) Heatmap of the top 50 differentially expressed genes. (C–D) Boxplots of the AMPK score and FAO-protective score, respectively. (E) Boxplot of PPARA expression. (F) Focused KEGG gene set enrichment analysis (GSEA) of representative AMPK-related and metabolism-associated pathways. For this supplementary analysis, the NAFLD group comprised NAFL and NASH samples, whereas healthy normal-weight samples were used as the Control group. Differential-expression analysis was performed using limma, and two-group comparisons in panels C–E were performed using the Wilcoxon rank-sum test.
