# Supplementary material for: Hepatoprotective Formula 919 ameliorates hepatic steatosis and metabolic disturbance in NAFLD with involvement of AMPK signaling
Source: Front Pharmacol. 2026 Sep 28;17:1892654. doi: 10.3389/fphar.2026.1892654 (PMC13654718; doi:10.3389/fphar.2026.1892654)
Supplement: Supplementary file 2 [file Table1.docx]

**Supplementary Table S1**

Database-specific search strategies, target-selection criteria, and access information for HF919- and NAFLD-related target retrieval.

| **Database** | **Purpose** | **Search entity/term** | **Species** | **Selection/processing criteria** | **Version(if available)/access date** |
| --- | --- | --- | --- | --- | --- |
| TCMSP | Compound-associated target retrieval | Cryptotanshinone; Licocoumarone; Schizandrin; Skullcapflavone II; Hydroxytanshinone | Homo sapiens | Human targets retained; official gene symbols; duplicate targets removed | v2.3; 10 Jan 2026 |
| CTD | Compound-associated target retrieval | Five representative HF919 constituents listed above | Homo sapiens | Human targets retained; official gene symbols; duplicates removed | 10 Jan 2026 |
| PharmMapper | Compound target prediction | Five representative HF919 constituents listed above | Homo sapiens | Human targets retained; official gene symbols; duplicates removed | 10 Jan 2026 |
| SwissTargetPrediction | Compound target prediction | Five representative HF919 constituents listed above | Homo sapiens | Human targets retained; official gene symbols; duplicates removed | 10 Jan 2026 |
| GeneCards | Disease-target retrieval | Non-alcoholic fatty liver disease (NAFLD) | Homo sapiens | Human disease-associated targets retained; official gene symbols; duplicates removed | 10 Jan 2026 |
| OMIM | Disease-target retrieval | Non-alcoholic fatty liver disease (NAFLD) | Homo sapiens | Human disease-associated targets retained; official gene symbols; duplicates removed | 10 Jan 2026 |
| PharmGKB | Disease-target retrieval | Non-alcoholic fatty liver disease (NAFLD) | Homo sapiens | Human disease-associated targets retained; official gene symbols; duplicates removed | 10 Jan 2026 |
| TTD | Disease-target retrieval | Non-alcoholic fatty liver disease (NAFLD) | Homo sapiens | Human disease-associated targets retained; official gene symbols; duplicates removed | 10 Jan 2026 |
| DrugBank | Disease-target retrieval | Non-alcoholic fatty liver disease (NAFLD) | Homo sapiens | Human disease-associated targets retained; official gene symbols; duplicates removed | 10 Jan 2026 |
| DisGeNET | Disease-target retrieval | Non-alcoholic fatty liver disease (NAFLD) | Homo sapiens | Human disease-associated targets retained; official gene symbols; duplicates removed | 10 Jan 2026 |
