# Supplementary material for: Hepatoprotective Formula 919 ameliorates hepatic steatosis and metabolic disturbance in NAFLD with involvement of AMPK signaling
Source: Front Pharmacol. 2026 Sep 28;17:1892654. doi: 10.3389/fphar.2026.1892654 (PMC13654718; doi:10.3389/fphar.2026.1892654)
Supplement: Supplementary file 3 [file Table2.docx]

**Supplementary Table S2**

Characteristics, expression-matrix sources, preprocessing procedures, and analytical use of the public liver transcriptomic datasets.

| **Dataset** | **Platform** | **Sample composition** | | **Expression-matrix source used for analysis** | | **Preprocessing / data processing** | **Analysis in this study** |
| --- | --- | --- | --- | --- | --- | --- | --- |
| **GSE126848** | GPL18573, Illumina NextSeq 500 | 57 human liver biopsies: healthy normal-weight, n=14; healthy obese, n=12;  NAFL, n=15; NASH, n=16 | | | Gene-symbol-by-sample expression matrix (SampleTypeMatrix.csv) derived from the GSE126848 expression data and used for the present analysis; the GEO record also provides the raw gene-count matrix (GSE126848_Gene_counts_raw.txt.gz). | Gene identifiers were standardized to uppercase gene symbols; entries with missing/blank gene symbols and genes with no valid expression values were removed; missing values within individual genes were median-imputed where applicable; duplicated gene symbols were collapsed by retaining the entry with the highest mean expression. Samples were assigned to the predefined Control and disease groups according to the sample labels. | Healthy normal-weight samples were used as the Control group. Control vs NASH was used as the primary comparison, whereas NAFL and NASH samples were combined as the NAFLD group for the supplementary Control vs NAFLD comparison. |
| **GSE130970** | GPL16791, Illumina HiSeq 2500 | 78 human liver biopsies: 6 histologically normal and 72 spanning the NAFLD spectrum (NAS 0–6; fibrosis stage 0–4) | GEO processed TPM matrix: GSE130970_all_sample_salmon_tximport_TPM_entrez_gene_ID.csv.gz | | | In the original GEO processing, transcript abundance was quantified using Salmon v0.9.1 against GRCh37/Ensembl release 75; gene-level TPM values were generated using tximport v0.99.8 with lengthScaledTPM. Samples were subsequently grouped according to the available histopathological annotations. | Group-wise comparisons of pathway scores and selected gene expression levels were performed across NAS, ballooning, steatosis, lobular inflammation, and fibrosis severity categories. |
