# Supplementary material for: Hepatoprotective Formula 919 ameliorates hepatic steatosis and metabolic disturbance in NAFLD with involvement of AMPK signaling
Source: Front Pharmacol. 2026 Sep 28;17:1892654. doi: 10.3389/fphar.2026.1892654 (PMC13654718; doi:10.3389/fphar.2026.1892654)
Supplement: Supplementary file 4 [file Table3.docx]

**Supplementary Table S3**

Receptor structures and receptor-specific docking-grid parameters used for molecular docking.

| **Target** | **PDB ID** | **Center x (Å)** | **Center y (Å)** | **Center z (Å)** | **Size x (Å)** | **Size y (Å)** | **Size z (Å)** |
| --- | --- | --- | --- | --- | --- | --- | --- |
| AMPK | 1Z0N | 5.123 | 7.518 | 4.479 | 44 | 64 | 62 |
| TGFB1 | 8UDZ | 21.698 | −37.832 | 53.597 | 76 | 84 | 116 |
| ARG1 | 1D3V | 9.597 | 20.720 | 1.840 | 58 | 48 | 52 |

Note: Grid-center coordinates and box dimensions are given in Å. Docking was performed using AutoDock Vina v1.2.3 with an exhaustiveness of 40, num_modes = 10, and energy_range = 5 kcal/mol.
